# Ndufs4 ablation decreases synaptophysin expression in hippocampus

**DOI:** 10.1038/s41598-021-90127-4

**Published:** 2021-05-26

**Authors:** Subrata Kumar Shil, Yoshiteru Kagawa, Banlanjo Abdulaziz Umaru, Fumika Nanto-Hara, Hirofumi Miyazaki, Yui Yamamoto, Shuhei Kobayashi, Chitose Suzuki, Takaaki Abe, Yuji Owada

**Affiliations:** 1 Department of Organ Anatomy, Tohoku University Graduate School of Medicine, 2-1 Seiryo-machi, Aoba-ku, Sendai, Miyagi 980-8575 Japan; 2 Division of Animal Metabolism and Nutrition, Institute of Livestock and Grassland Science, National Agriculture and Food Research Organization, Tsukuba, 305-0901 Japan; 3 Department of Anatomy, Tohoku Medical and Pharmaceutical University, Sendai, 981-0905 Japan; 4 Department of Nephrology, Endocrinology and Vascular Medicine, Tohoku University Graduate School of Medicine, Sendai, 980-8575 Japan

**Keywords:** Molecular biology, Neuroscience

## Abstract

Altered function of mitochondrial respiratory chain in brain cells is related to many neurodegenerative diseases. NADH Dehydrogenase (Ubiquinone) Fe-S protein 4 (Ndufs4) is one of the subunits of mitochondrial complex I and its mutation in human is associated with Leigh syndrome. However, the molecular biological role of Ndufs4 in neuronal function is poorly understood. In this study, upon Ndufs4 expression confirmation in NeuN-positive neurons, and GFAP-positive astrocytes in WT mouse hippocampus, we found significant decrease of mitochondrial respiration in Ndufs4-KO mouse hippocampus. Although there was no change in the number of NeuN positive neurons in Ndufs4-KO hippocampus, the expression of synaptophysin, a presynaptic protein, was significantly decreased. To investigate the detailed mechanism, we silenced Ndufs4 in Neuro-2a cells and we observed shorter neurite lengths with decreased expression of synaptophysin. Furthermore, western blot analysis for phosphorylated extracellular regulated kinase (pERK) revealed that Ndufs4 silencing decreases the activity of ERK signalling. These results suggest that Ndufs4-modulated mitochondrial activity may be involved in neuroplasticity via regulating synaptophysin expression.

## Introduction

Mitochondria are subcellular organelles that play vital roles in the generation of cellular energy in the form of ATP via oxidative phosphorylation, synthesis of metabolic precursors for macromolecules, calcium buffering and programmed cell death^1–3^. Given these essential functions, mitochondrial dysfunction in tissues with high energy demand can lead to a wide variety of diseases^4^. In the CNS, mitochondrial function is crucial for neurons because neurons demand high levels of ATP for cellular function such as synaptic neurotransmission and plasticity^5,6^. Furthermore, mitochondria provide a buffering machinery to regulate calcium concentration during signal transduction in neurons^7^. They are also involved in biosynthesis of iron-sulfur and heme in neurons for neurotransmitter synthesis in synapses^8^. Thus, altered mitochondrial function may lead to neurodegenerative diseases such as Parkinson's disease (PD), Alzheimer's disease (AD), Huntington's disease, amyotrophic lateral sclerosis and Leigh syndrome^9,10^. For example, it has been reported that progressive accumulation of mitochondrial amyloid-*β* (A*β*) leads to mitochondrial dysfunction resulting in neuronal damage and cognitive decline in patients and transgenic mouse model of AD^11^. In another study, embryonic neurons derived from transgenic AD mouse hippocampus exhibited significantly decreased mitochondrial respiration^12^. Moreover, mitochondrial complex I deficiency has been detected in the substantia nigra and frontal cortex tissue of PD patients^13^. Recently, excitatory neurons isolated from the pluripotent stem cells of MELAS (mitochondrial encephalomyopathy, lactic acidosis and stroke-like episodes) patient showed impaired mitochondrial function with delayed neural maturation, reduced dendritic complexity and fewer functional excitatory synapses^14^. Thus, understanding the role of mitochondrial function in the development of neurodegenerative disease is quite essential.

Mitochondrial complex I (NADH: ubiquinone oxidoreductase) is the main gateway for electron entry to the electron transport chain (ETC)^15^. NADH Dehydrogenase (Ubiquinone) Fe-S protein 4 (Ndufs4), one of the subunits in mitochondrial complex I is involved in the assembly and stability of Complex I^16^. The gene encoding human NDUFS4 is a hot spot for fatal pathogenic mutations that commonly results in Leigh syndrome^17^, an infantile onset, progressive neurodegenerative disorder clinically characterized by motor and intellectual retardation, seizures, respiratory insufficiency and failure to thrive leading to early death^18,19^. Similarly, global Ndufs4-KO mouse showed retarded growth, loss of motor ability, breathing abnormalities, and death^20^. Moreover, previous studies have demonstrated the importance of Ndufs4 in neuronal function. In recent study, glutamatergic neuron specific Ndufs4 loss in mice showed a significant impairment of neuronal firing, motor deficit, brain stem inflammation and region specific marked astrogliosis^21^. Furthermore, GABAergic neurons specific Ndufs4 loss exhibited basal ganglia inflammation and severe epileptic seizures preceding death^21^. Furthermore, dopaminergic neuron specific Ndufs4-KO mouse showed the impairment of dopamine release in striatum, amygdala without loss of neuron^22–24^. These evidence suggest that Ndufs4 loss in neurons might be a key factor for the development of encephalopathy. However, the molecular biological role of Ndufs4 in neural function is still unknown.

In this study, we explored how Ndufs4 ablation affects neuron function focusing on neurite growth and synaptic plasticity and found that synaptophysin, a presynaptic protein, expression decreases significantly with a decreased activity of ERK signalling. This novel finding may provide more insights in hippocampal pathophysiology of neurodegenerative disease and may be useful in designing an effective treatment strategy.

## Results

### Expression and localization of Ndufs4 in cerebral cortex and hippocampus

**Figure 1:**
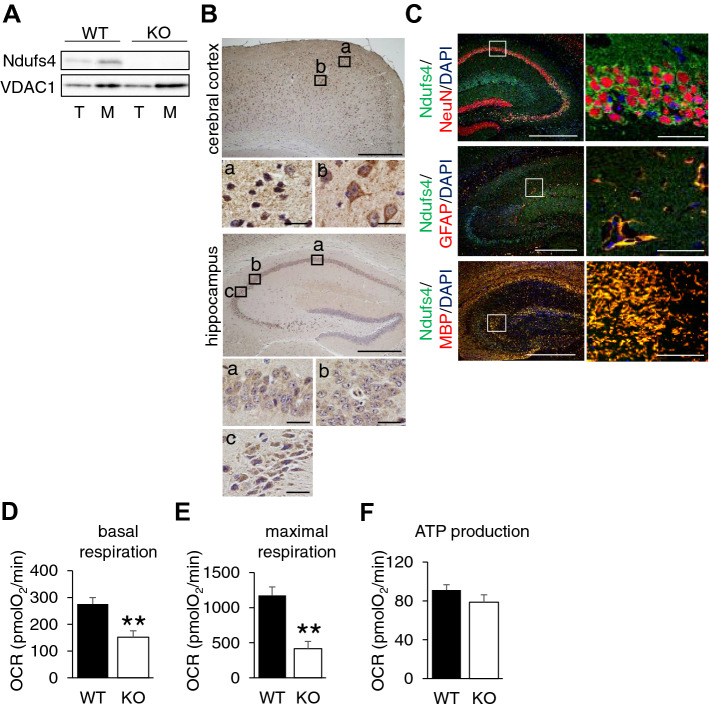
Expression and localization of Ndufs4 in cerebral cortex and hippocampus. (**A**) Representative image from Western blot for Ndufs4 in brain of WT and Ndufs4-KO mouse. T: total protein, M: protein from isolated mitochondria. (**B**) Representative image from enzyme-based immunohistochemistry showing the expression of Ndufs4 in cerebral cortex and hippocampus. Insets enlarge the boxed area. Scale for low magnification: 500 µm, for high magnification: 50 µm. (**C**) Representative image from co-immunofluorescence staining of Ndufs4 (Green) with NeuN, GFAP and MBP (red) in hippocampus of WT mouse. Boxed area is enlarged on right. Scale for low magnification: 500 µm, for high magnification: 40 µm. (**D**–**F**) Mitochondrial complex I respiration assay by XF24 analyser using isolated mitochondria from punch out hippocampus of WT (n = 3) and Ndufs4-KO (n = 3) mouse.

We first explored Ndufs4 expression and localization in the brain. Western blot analysis using isolated mitochondria from brain confirmed Ndufs4 expression (Fig. 1A). To determine the expression level in different brain regions, we punched out the tissue from the cerebral cortex, hippocampus, and cerebellar cortex. qPCR analysis demonstrated that Ndufs4 was expressed in all regions of the brain although these expression levels were lower compared to the expression in the heart tissue (Supplemental Fig. 1A,B). Furthermore, enzyme-based immunohistochemistry also confirmed the expression of Ndufs4 in cerebral cortex and CA1, CA2 and CA3 region of hippocampus (Fig. 1B). To investigate which cell types express Ndufs4, we performed co-immunostaining of Ndufs4 with several cell markers such as neuronal nuclei (NeuN) for neuron, glial fibrillary acidic protein (GFAP) for astrocyte and myelin basic protein (MBP) for oligodendrocyte. We found that Ndufs4 co-localized with all cell markers in hippocampus and cerebral cortex with equal fluorescence (Fig. 1C and Supplemental Fig. 1C–F). Then, to explore the mitochondrial functionality, we measured complex I respiration using the isolated mitochondria from punch out hippocampus tissue and found a significant decrease in basal respiration and maximal respiration in Ndufs4-KO compared to WT, but ATP production was not changed (Fig. 1D–F).

### Elevated GFAP expression was observed in Ndufs4-KO hippocampus, but not in primary cultured Ndufs4-KO astrocytes

**Figure 2:**
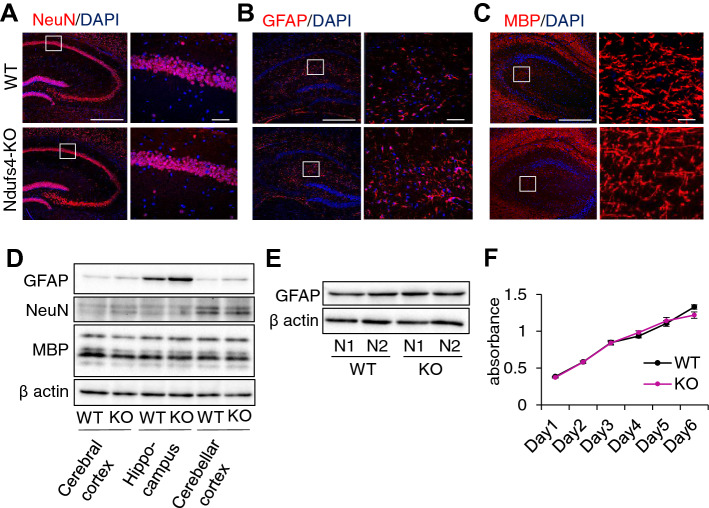
Ndufs4 deficiency shows astrocyte reactivity in hippocampus displaying normal GFAP expression and proliferation in primary culture of astrocyte. (**A**–**C**) Representative image from immunofluorescence staining of NeuN (**A**), GFAP (**B**) and MBP (**C**) in hippocampus of WT and Ndufs4-KO mouse. Boxed area is enlarged on right. Scale for low magnification: 500 µm, for high magnification: 50 µm. (**D**) Representative image of Western blot for GFAP, NeuN and MBP in different regions of WT and Ndufs4-KO mouse brain. (**E**) Representative image of Western blot for GFAP in WT and Ndufs4-KO primary astrocytes. (**F**) Proliferation assay of WT (n = 6) and Ndufs4-KO (n = 6) astrocytes.

To evaluate whether Ndufs4 deficiency alters the expression of cell markers and cell population, immunostaining and Western blot were performed using hippocampus of WT and Ndufs4-KO mice. Although there was no difference in expression of NeuN-positive neuron and MBP-positive oligodendrocyte (Fig. 2A,C,D), interestingly, an increased number of hypertrophic GFAP-positive astrocytes with increased number of processes were found in Ndufs4-KO hippocampus (Fig. 2B; Supplemental Fig. 2A,B). Elevated GFAP expression in Ndufs4-KO hippocampus was also confirmed by Western blot (Fig. 2D; Supplemental Fig. 2C). This led us to hypothesize that reactive gliosis, characterized by hypertrophy of the astrocyte cellular processes and high levels of expression of GFAP, may be occurring in Ndufs4-KO brain. As such, to investigate whether Ndufs4 deficiency in astrocytes affects the function of astrocytes, we prepared primary cultured astrocytes from Ndufs4-KO brain (Supplemental Fig. 2D), but we could not find gliosis character such as increased GFAP expression and increased cell proliferation (Fig. 2E,F; Supplemental Fig. 2E) in Ndufs4-KO astrocytes, suggesting that the gliosis character observed in Ndufs4-KO mouse brain may be due to altered neuroplasticity in hippocampus.

### Ndufs4 deficiency decreased synaptophysin expression in hippocampus

**Figure 3:**
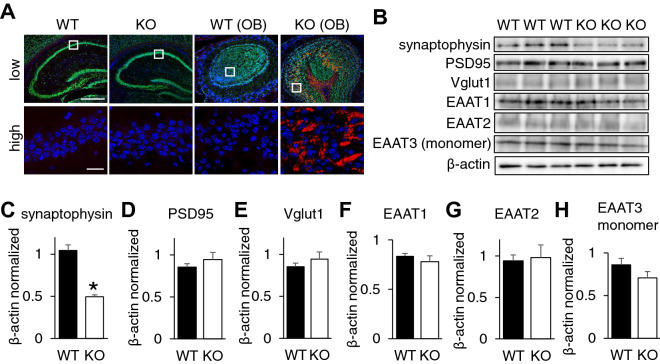
Ndufs4 deficiency shows altered synaptic protein expression without neuronal loss. (**A**) Representative image of co-immunofluorescence staining of NeuN and Caspase 3 in hippocampus and olfactory bulb (OB) of WT mouse and Ndufs4-KO. Insets enlarge the boxed area. Low magnification green indicates NeuN and red indicates caspase 3, scale bar: 400 µm. In high magnification red indicates caspase 3 with no green stain, scale: 20 µm. (**B**) Representative images of Western blot for synaptophysin, PSD95, Vglut1, EAAT1, EAAT2, EAAT3 in WT and Ndufs4-KO mouse hippocampus. (**C**–**H**) Analysis of band densities by ImageJ (n = 3).

To ascertain whether any neuronal dysfunction occurred in hippocampus due to Ndufs4 deficiency, we counted the number of NeuN positive cells in hippocampus, but we did not find a significant difference between WT and Ndufs4-KO mouse brain (Supplemental Fig. 3A). Because previous reports have demonstrated the presence of neuron apoptosis in the olfactory bulb of Ndufs4-KO mouse^20^, supportively, we evaluated neuron apoptosis, but we could not find cleaved caspase 3 positive cells in both WT and Ndufs4-KO hippocampus (Fig. 3A). Then, we investigated the synaptic protein expression in hippocampus because mitochondrial function is closely associated with the synthesis of synaptic protein^25^. Notably, synaptophysin expression was significantly decreased in Ndufs4-KO hippocampus (Fig. 3B,C). However, there was no change in the expression of other proteins including PSD95, Vglut1, EAAT1, EAAT2 and EAAT3 (Fig. 3B and D–H).

### Knockdown of Ndufs4 impairs neurite outgrowth and synaptophysin-positive puncta in differentiated Neuro-2a cell

**Figure 4:**
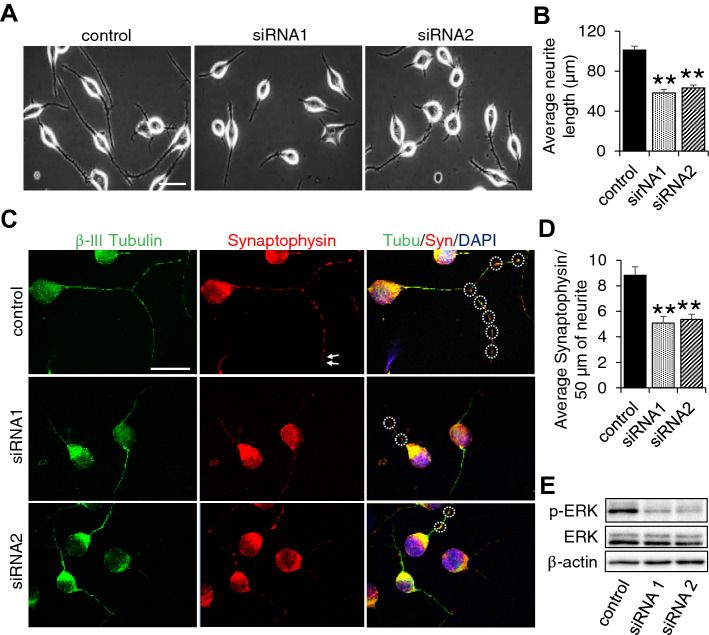
Ndufs4 silencing impairs neurite outgrowth and synaptophysin-positive puncta in differentiated Neuro-2a cell. (**A**) Representative phase contrast image of neurite outgrowth in Ndufs4 silenced differentiated Neuro-2a cells. Scale bar 50 µm. (**B**) ImageJ analysis of average neurite length of differentiated cells (n = 6). (**C**) Representative image of differentiated Neuro-2a cells immunostained for β-III tubulin and synaptophysin. Circles indicate location of synaptophysin in neurites. Scale bar 40 µm (**D**) ImageJ analysis of average number of synaptophysin in neurites/50 µm (n = 6). (**E**) Representative image of Western blot for the pERK and ERK in control and Ndufs4 silenced differentiated Neuro-2a cells.

To further investigate the role of Ndufs4 in synaptic change, we silenced Ndufs4 in Neuro-2a cells by siRNA treatment, then induced differentiation by treatment with retinoic acid (Supplemental Fig. 4A–C). We also confirmed that Ndufs4 silencing impaired mitochondrial function including basal respiration, maximum respiration, as well as ATP production (Supplemental Fig. 4D). Using these cells, we found that Ndufs4 silencing decreased the average neurite length compared to control (Fig. 4A,B) along with the number of synaptophysin puncta per length of β-III tubulin positive neurite (Fig. 4C,D). Furthermore, Ndufs4 silencing significantly decreased the expression level of phosphorylated-ERK signalling (Fig. 4E; Supplemental Fig. 4E). To confirm the direct relation between ERK activity and neurite growth/synaptophysin expression, we performed a rescue experiment using a pERK agonist Carbamylcholine Chloride (carbachol). We found a significantly higher neurite growth and synaptophysin expression in carbachol treated Ndufs4 knockdown cells, and even in control cells (Supplemental Fig. 5A–D). Considering that carbachol is a nonspecific cholinergic agonist and acts on nicotinic and muscarinic receptors, and as well, activates many downstream signalling pathways^26–28^, it may activate other cell signalling outside of the Ndufs4 cascade, but there is a strong possibility that drug-induced-increased pERK activity in Ndufs4 knockdown cells partially rescued neurite growth and synaptophysin expression. Taken together, these data suggest that Ndufs4-modulated mitochondrial function may be involved in neuroplasticity through the ERK signalling followed by alteration of neurite growth and synaptophysin expression.

## Discussion

Mitochondrial function in neuron is quite essential for electrical activity, axon extension, regeneration, branching, synaptic formation, and synaptic neurotransmission^5,29,30^, and altered mitochondrial function leads to neurodegenerative diseases^31–33^. Ndufs4 is the gene responsible for Leigh syndrome^17^. It has been reported that loss of Ndufs4 reduces maximum CI dependent oxidative phosphorylation in synaptosomal mitochondria of olfactory bulb, brainstem and cerebellum^33^. Furthermore, striatal medium spiny neurons specific Ndufs4 loss progressively impairs the motor function without loss of neuronal number^22^. However, the molecular function of Ndufs4 in neuron is still not well deciphered. In this study, we first demonstrated Ndufs4 expression in hippocampal neurons in WT and then showed mitochondrial dysfunction with decreased expression of synaptophysin without loss of neuronal cells in Ndufs4 deficient mouse hippocampus.

Mitochondria are intimately involved in cell signalling pathways such as growth factor signalling, differentiation, cell death signalling, and autophagy through regulating the levels of intracellular signalling molecules such as Ca^2+^, ATP and ROS^34^. Notably, mitochondrial dysfunction promotes p38 mitogen-activated protein kinase activity, resulting in neuronal intracellular responses including inflammation and apoptosis^35–37^. Similarly, decreased activity of ERK signalling inhibits mitochondrial fusion and stimulates apoptotic mitochondrial membrane permeabilization through phosphorylation of mitochondrial fusion protein, mitofusin 1, in rat primary cortical neurons thereby regulating cell death^38^. Furthermore, pharmacological inhibition of c-Jun N-terminal kinases results in a decreased mitochondrial membrane potential in motor neurons, leading to cell degeneration^39^. These previous studies thus suggest the involvement of mitochondria in the cell signalling cascade. In this study, although there was no difference in the phosphorylation level of ERK between WT and Ndufs4 KO hippocampus (Supplemental Fig. 4F,G), silencing of Ndufs4 decreased the levels of phosphorylation of ERK. These results may reflect the close association between Ndufs4 in neuron and ERK signalling.

It is well known that intracellular cell signalling is responsible for presynaptic formation. For example, altered ERK signalling has been shown to impair synaptic plasticity in the hippocampus^40–42^. It has also been reported that ERK is responsible for the presynaptic modulation of synaptic plasticity in the hippocampal CA3 region that requires synapsin 1, a presynaptic protein^43^. Similarly, another study also demonstrated that pharmacological blockade of ERK activity prevents stimulation-induced dendritic spine formation in hippocampal CA1 pyramidal neuron in ex vivo slice systems^44^. In this study, we demonstrated a decreased activity of ERK signalling and decrease of synaptophysin expression, suggesting that Ndufs4 is regulating the synaptophysin expression through modulating ERK signalling. Furthermore, a rescue experiment with carbachol demonstrated that a significantly higher neurite growth and synaptophysin expression in carbachol treated Ndufs4 knockdown cells, and even in control cells. Considering that carbachol is a nonspecific cholinergic agonist and acts on nicotinic and muscarinic receptors, and as well, activates many downstream signalling pathways^26–28^, it may also activate other cell signalling outside of the Ndufs4 cascade, but there is a strong possibility that drug-induced-increased pERK activity in Ndufs4 knockdown cells partially rescued the synaptophysin expression. Further experiments using a selective ERK agonist such a mutant of ERK will clarify this issue.

Reactive gliosis is commonly observed with neuron loss or dysfunction in CNS disorders. Previous research shows a marked gliosis with significant loss of synapse in cerebral cortex of Alzheimer’s patients^45^. Similarly, another study shows astroglial reactivity in hippocampus in a mouse model of traumatic axonal injury^46^. Notably, it has been reported that glutamatergic neuron specific Ndufs4-KO mouse shows elevated GFAP expression in vestibular nucleus, cerebellar fastigial nucleus and inferior olive^21^. Similarly, GABAergic neuron specific Ndufs4-KO mouse shows elevated GFAP in basal ganglia, substantia nigra pars reticulata and olfactory bulb^21^. Furthermore, Nestin-driven Ndufs4-KO mouse shows elevated GFAP in olfactory bulb, cerebellum, and vestibular nuclei^20^ suggesting neuron dysfunction may trigger astrogliosis. In this study, gliosis character was observed in in vivo Ndufs4-KO hippocampus with decreased synaptophysin expression with no neuron loss. In addition, primary cultured astrocytes from Ndufs4-KO hippocampus did not show any gliosis character. These results indicate that altered neuroplasticity including impaired synaptic formation may be the inducing factor of gliosis. Further detailed study may reveal the mechanism underlying the astrocyte reactivity in Ndufs4-KO mouse.

In summary, we provide new insights on the effect of Ndufs4 loss in the function of hippocampal neurons. Our work highlighted the involvement of Ndufs4 in neuroplasticity by regulating synaptophysin expression through phosphorylation of ERK. This knowledge may be useful in understanding the pathophysiological aspect of neurodegenerative diseases but, further clear understanding of molecular mechanism of Ndufs4 is quite essential.

## Materials and methods

### Animals

Ndufs4-KO mice (B6.129S4-Ndufs4^tm1.1Rpa^/J) were purchased from The Jackson Laboratory (Maine, USA). The animals were housed in standard cages in a temperature and humidity-controlled room with a 12 h light–dark cycle and ad libitum access to food and water. Animal experiments were carried out in compliance with the ARRIVE guidelines (Animal Research: Reporting of In Vivo Experiments). All animal care procedures were approved by the Animal Care and Use Committee of Tohoku University Graduate School of Medicine and under the law and notification of the Japanese government. Efforts were taken to minimize animal sufferings. The heterozygous mice were inter-crossed to get the homozygous KO mouse pups and then genotyped to identify the individual mouse.

### Tissue collection and quantitative real-time PCR

The brain was removed quickly under anaesthesia and made the serial coronal sections, followed by punching out the hippocampal tissue. Total RNA was extracted by RNeasy Mini Kit (Qiagen, Hilden, Netherlands). Transcriptor High Fidelity cDNA Synthesis Kit (Roche, Basel, Switzerland) was used for reverse transcription. qPCR for *Ndufs4* was performed using StepOnePlus Real-Time PCR system (Applied Biosystems, California, USA) using following mouse specific TaqMan Probes: *Ndufs4*:Mm0656176_ml, *Actb*: Mm02619580_gl. *Actb* was used as endogenous control. Relative gene expression was calculated using the 2^−∆∆Ct^ method.

### Immunostaining

The male mice at p21 were transcardially perfused with glyoxal (ALTFiX, Falma Co., Ltd. Tokyo, Japan) under anaesthesia. Brains were removed, post-fixed in glyoxal for overnight at 4 °C and subsequent dehydration by gradient concentration of alcohol, cleaned by xylene, infiltrated, and embedded in paraffin. Brain samples were cut in a serial 4 μm of coronal sections using a sliding microtome (Leica Biosystems, Wetzlar, Germany).

For enzyme-based immunohistochemistry, the sections were deparaffinized, rehydrated in phosphate buffered saline (PBS) and permeabilized with 0.3% Triton X-100 in PBS, blocked in 5% fetal bovine serum (FBS) followed by overnight incubation of primary antibody shown in Table 1. After primary antibody incubation, sections were washed with PBS and incubated with biotin conjugated goat anti mouse IgG. Then, sections were incubated with ABC reagent (VECTASTAIN Elite ABC kit, Vector Laboratories Inc., Cat. No. PK-6100, California, USA). Haematoxylin stain was used as nuclear counterstaining.

**Table 1:** List of primary and secondary antibodies.

1st Antibody	Dilution	Vendor/reference	2nd Antibody/dilution
Rabbit polyclonal anti-Ndufs4	WB: 1:500	Sigma Aldrich, Cat. No. HPA003884	Goat anti-rabbit IgG-HRP conjugated (1:2000)
Mouse monoclonal anti-Ndufs4	IF: 1:50	SantaCruz, Cat. No. sc-100567	Goat anti-mouse IgG-Alexa 488 (1:500)
Rat monoclonal anti-GFAP	WB: 1:2000, IF: 1:200	Thermo Fisher Scientific, Cat. No.13-0300	Goat anti-rat IgG-HRP conjugated (1:2000), Goat anti-rat IgG-Alexa 568 (1:500)
Mouse monoclonal anti NeuN	WB: 1:1000 IF: 1:200	Chemicon, Cat No. MAB377	Goat anti-mouse IgG-HRP conjugated (1:2000) Goat anti-mouse IgG-Alexa 488 (1:500)
Rabbit monoclonal anti-NeuN	IF: 1:200	Cell Signaling, Cat. No. 24307	Goat anti-rabbit IgG-Alexa 568 (1:500)
Rat monoclonal anti MBP	WB: 1:1000, IF: 1:200	Abcam, Cat. No. ab7349	Goat anti-rat IgG-HRP conjugated (1:2000), Goat anti-rat IgG-Alexa 568 (1:500)
Rabbit polyclonal anti PSD95	WB: 1:2000 IF: 1:200	Cell Signaling, Cat. No. 3450)	Goat anti-rabbit IgG-HRP conjugated (1:2000) Goat anti-rabbit IgG-Alexa 568 (1:500)
Mouse monoclonal anti Synaptophysin	WB: 1:400, IC:1:100	Sigma Aldrich, Cat. No. S5768	Goat anti-mouse IgG- HRP conjugated (1:2000), Goat anti-mouse IgG-Alexa 488 (1:250)
Rabbit mococlonal anti-β-III Tubulin	IC: 1:00	Cell Signaling, Cat. No. 5666P	Goat anti-rabbit IgG-Alexa 568 (1:500)
Rabbit polyclonal anti EAAT1	WB: 1:1000	Cell Signaling, Cat. No. 4166	Goat anti-rabbit IgG-HRP conjugated (1:2000)
Rabbit polyclonal anti EAAT2	WB: 1:1000	Abcam, Cat. No. ab41621	Goat anti-rabbit IgG-HRP conjugated (1:2000)
Rabbit polyclonal anti-Rat EAAC1 (EAAT3)	WB: 1:1000	Alpha Diagnostic International, Cat. No. EAAC11-S	Goat anti-rabbit IgG-HRP conjugated (1:2000)
Guinea pig polyclonal anti Vglut 1	WB: 1:1000	EMD Millipore, Cat. No. AB5905	Goat anti-guinea pig IgG-HRP conjugated (1:2000)
Rabbit polyclonal anti p44/42 MAPK (Erk1/2)	WB: 1:1000	Cell Signaling, Cat. No. 9102	Goat anti-rabbit IgG-HRP conjugated (1:2000)
Rabbit polyclonal anti phospho-p44/42 MAPK (Erk1/2)	WB: 1:1000	Cell Signaling, Cat. No. 9101	Goat anti-rabbit IgG-HRP conjugated (1:2000)
Mouse monoclonal anti-β-actin	WB: 1:2000	Santa Cruz, Cat. No. sc-47778	Goat anti-mouse IgG-HRP conjugated (1:2000)
Rabbit Polyclonal anti-VDAC1	WB: 1:1000	Abcam, Cat. No. ab15895	Goat anti-rabbit IgG-HRP conjugated (1:2000)
Rabbit polyclonal anti cleaved caspase 3	IF: 1:300	Cell Signaling, Cat. No. 9661	Goat anti-rabbit IgG-Alexa 568 (1:500)

For immunofluorescence, sections were deparaffinised, hydrated followed by permeabilization with 0.3% Tritox X-100. Sections were incubated in primary antibodies shown in Table 1. Respective secondary antibodies and DAPI (Thermo Fisher Scientific Inc., Massachusetts, USA) reaction were allowed. Images were acquired using a confocal laser scanning microscope (LSM780; Carl Zeiss, Oberkochen, Germany). Number of astrocytes and astrocyte processes were counted using cell counter plugin of ImageJ (NIH, USA). Actual fluorescence intensity was measured after subtracting the background intensity using ImageJ software.

For immunofluorescence using cryosection, brain was removed, post fixed with 4% paraformaldehyde (PFA) and cryoprotected in 15% sucrose followed 30% sucrose in PBS until the tissue sink. Then a cryostat (Tissue-Tek OCT Compound, Sakura Finetek, Cat. No. 4583, Osaka, Japan) was used to cut 14 µm section. Sections were blocked with 20% FBS in 0.3% Triton X-100 in PBS for 1 h followed by overnight incubation of primary antibody (Table1) in 4 °C. Respective secondary antibodies were used, and images were acquired using a confocal laser scanning microscope (LSM780).

Immunocytochemistry of synaptophysin in Neuro-2a cells neurites was performed according to described previously^47^. Cells were fixed with 4% PFA and permeabilized with 0.1% Triton X-100 and incubated by primary antibodies shown in Table 1. Respective secondary antibody with DAPI reaction was allowed. Images were captured by confocal laser scanning microscope LSM780. Synaptophysin stained red puncta was counted as presynapse and respective neurite length was measured using ImageJ software.

### Purified astrocyte culture

Primary astrocytes were prepared from cerebral cortices and hippocampi of 0- to 1- day-old WT and littermate Ndufs4-KO mice as described previously^48^. Briefly, the cerebral cortices and hippocampi were isolated carefully with removing the olfactory bulbs and the meninges. Tissues were transferred in Dulbecco’s Modified Eagle’s Medium (DMEM) (Sigma Aldrich, Missouri, USA) supplemented with 10% FBS, 20 mM d-glucose, 1% v/v penicillin/streptomycin and treated with trypsin (Life Technologies, California, USA) at a final concentration of 0.25% for 20 min in 37 °C. The harvested cells were resuspended in DMEM containing 10% v/v heat-inactivated FBS, 1% v/v penicillin/streptomycin and filtered using a 100 μm cell strainer (Falcon, New York, USA). Finally, the cells from individual brain were seeded in respective T-75 flasks (BD Falcon, Schaffhausen, Switzerland). Medium was replaced every three days interval. After 11–12 days, the cells reached confluency and the flasks were shaken for 24 h at 200 rpm to remove microglia and oligodendrocyte progenitor cells. Following a recovery day, the remaining astrocytes on the adherent monolayer were detached using 0.1% w/v trypsin with 0.02% w/v of ethylenediaminetetraacetic acid (EDTA) and passaged in dishes or plates for further analysis.

### Isolation of mitochondria from tissue and cultured cells

Mitochondria were isolated from brain and heart as described before^49^ with modifications. Briefly, isolated brain and heart were trypsinized for 20 min with shaking for every 5 min. Tissue was resuspended in mitochondrial isolation buffer (0.25 M sucrose, 20 mM Tris–HCl (pH 8.0), 0.1 mM EDTA (pH 8.0) and homogenized by Dounce homogenizer with tight pestle with gentle 20 strokes. Homogenate was allowed for centrifugation at 600×*g* for 10 min at 4 °C. Supernatant was transferred carefully and centrifuged again at 12,000×*g* for 15 min at 4 °C. Finally, the pellet was dissolved in sodium dodecyl sulfate polyacrylamide gel electrophoresis (SDS-PAGE) sample buffer containing protease inhibitor (Roche). Protein concentration was measured by Pierce BCA Protein Assay Kit (Thermo Fisher Scientific Inc.) in accordance with manufacturer’s instructions.

### Isolation of hippocampal mitochondria and mitochondrial complex I respiration assay

Functional mitochondria were isolated according to previously published report^49^ with modifications. Briefly, hippocampal tissue was collected, chopped, and washed with Dulbecco's Phosphate-Buffered Saline followed by trypsinization. Then, mitochondria were isolated in ice cold isolation buffer (Sucrose 200 mM, Tris-MOPS 10 mM, EGTA/Tris 1 mM, final pH 7.4) using Dounce homogenizer. Homogenates were centrifuged firstly at low speed and then high speed to get the functional mitochondrial pellet. Complex I dependent mitochondrial respiration was measured using the seahorse XF24 extracellular flux analyzer (Agilent Technologies, California, USA) according to the manufacturer’s protocols and previous reports^22,33^. A total of 25 µg of mitochondria was seeded in XF24 cell culture plate with assay medium for complex I (Sucrose 70 mM, D (−) Mannitol 220 mM, KH_2_PO_4_ 10 mM, MgCl_2_ 5 mM, HEPES–KOH 2 mM, EGTA-KOH 1 mM, fatty acid free bovine serum albumin (BSA) 0.2%, D-Malic acid 5 mM and Sodium pyruvate 10 mM, pH 7.2) and centrifuged at 2000×*g* for 20 min at 4 °C. OCR was monitored by sequential injections of Adenosine diphosphate (ADP), Oligomycin, Carbonyl cyanide-4-(trifluoromethoxy)phenylhydrazone (FCCP) and Antimycin A to a final concentration of 4.25 mM, 2.5 μM, 5 µM and 4 μM in complex I assay medium respectively.

### Mitochondrial respiration assay for Ndufs4 silenced differentiated Neuro-2a

Oxygen consumption rate (OCR) of Neuro-2a cells was measured according to the manufacturer’s protocols and previous published article^50^. Briefly, transfected cells were seeded in XF24 culture plate, induced differentiation and cultured in assay medium containing 25 mM glucose, 2 mM L-glutamine, 1 mM sodium pyruvate and 30 mM NaCl (pH 7.4). Mitochondrial OCR was measured with sequential injection of Oligomycin, FCCP and rotenone with antimycin A through the ports in assay cartridge to a final concentration of 1 μg/ml, 1 μM and 5 μM, respectively. This allowed determination of the basal level of oxygen consumption, level of ATP production and maximal respiration.

### Western blot

Protein samples were separated on SDS-PAGE and then transferred to a polyvinylidene fluoride (PVDF) membrane (Merck Millipore, Massachusetts, USA). Membrane was blocked with 5% BSA in Tris–buffered saline with Tween 20 (T-TBS) and incubated with primary antibody shown in Table 1. The protein was visualized by an enhanced chemiluminescence. Images were acquired with ChemiDoc MP Imaging System (Bio-Rad Laboratories, California, USA). Anti-β-actin and anti-VDAC1 were used as probe to ensure the equal loading of the samples. Original full-length blots/gels are shown in Supplemental Figures.

### Astrocyte proliferation assay

WT and Ndufs4-KO primary astrocytes were seeded in 24 well plate at a density of 100,000 cells/well. Fifty microliters of Cell Count Reagent SF (Nacalai Tesque, Cat. No. 07553-44, Kyoto, Japan) was added to each well containing 500 µl of culture medium and the plates were incubated for one hour at 37 °C. Absorbance was recorded at 450 nm using the FlexStation 3 Multi-Mode Microplate Reader (Molecular Devices, California, USA).

### Ndufs4 silencing in Neuro-2a cells

Mouse neuroblastoma Neuro-2a cells were transfected with Ndufs4 siRNA (Thermo Fisher Scientific Inc., Cat. No. 10620318) using Lipofectamine RNAiMAX reagent (Thermo Fisher Scientific Inc., Cat. No. 13778-150) according to manufacturer’s protocol. Negative control medium GC duplex (Thermo Fisher Scientific Inc, Cat. No. 465372) was used for the control. Following mouse specific primer sequences were used: siRNA1: Ndufs4MSS275954(3_RNAI) 5′–3′ GAG AAA CUG GAU AUC ACA ACU UUA A, siRNA2: Ndufs4MSS275955(3_RNAI) 5′–3′ CAG AAU CUU UGU UCC UGC UCG CAA U.

### Evaluation of neurite outgrowth and presynapse number

Cells were passaged after 18 h of Ndufs4 siRNA transfection. To stimulate differentiation, the cells were then incubated in medium containing 20 µM of all-trans-Retinoic acid (RA) (Sigma Aldrich, Cat. No. R2625, Tokyo, Japan) for 18 h. Live cell images were captured by phase contrast microscope. The number of neurites in the individual cells were counted and lengths were measured as the distance from the centre of the cell soma to the tip of the neurite as reported previously^51^. Six random fields were examined from each siRNA, giving a total field of 18 and cell count was at least 70 cells/well. Each data point represents the mean of three individual wells in one experiment, and each experiment was repeated three times.

### pERK agonist carbachol treatment in Neuro-2a cells

Neuro-2a cells were seeded at a concentration of 1 × 10^5^ cells/ml, transfected with Ndufs4 siRNA followed by induction of differentiation using RA for 12 h in serum free medium. After 36 h of transfection, media containing RA was washed with DMEM medium and treated by serum free medium containing pERK agonist Carbamylcholine Chloride (carbachol) (FUJIFILM Wako Pure Chemical Corporation, Cat. No. 036-09841, Osaka, Japan) at a final concentration of 100 µM for 8 h. Images were taken and cells were fixed by 4% followed by co-immunofluorescence staining.

### Statistical analysis

Data were presented as mean ± SEM (standard error of mean) of at least six independent experiments with at least three biological replicates. Comparisons were made by two tailed unpaired Student’s t test. Differences were considered significant at P values < 0.05. Analysis was performed using the Microsoft Excel.

## Supplementary Information

Supplementary Information.

## References

[1] Perier C, Vila M (2012). Mitochondrial biology and Parkinson’s disease. Cold Spring Harb. Perspect. Med..

[2] Spinelli JB, Haigis MC (2018). Cellular metabolism. Nat. Cell Biol..

[3] Wang C, Youle RJ (2009). The role of mitochondria in apoptosis. Annu. Rev. Genet..

[4] Vakifahmetoglu-Norberg H, Ouchida AT, Norberg E (2017). The role of mitochondria in metabolism and cell death. Biochem. Biophys. Res. Commun..

[5] Rangaraju V, Calloway N, Ryan TA (2014). Activity-driven local ATP synthesis is required for synaptic function. Cell.

[6] Supplie LM (2017). Respiration-deficient astrocytes survive as glycolytic cells in vivo. J. Neurosci..

[7] Wang W, Zhao F, Ma X, Perry G, Zhu X (2020). Mitochondria dysfunction in the pathogenesis of Alzheimer’s disease: Recent advances. Mol. Neurodegener..

[8] Mena NP, Urrutia PJ, Lourido F, Carrasco CM, Núñez MT (2015). Mitochondrial iron homeostasis and its dysfunctions in neurodegenerative disorders. Mitochondrion.

[9] Johri A, Beal MF (2012). Mitochondrial dysfunction in neurodegenerative diseases. J. Pharmacol. Exp. Ther..

[10] Panchal K, Tiwari AK (2019). Mitochondrial dynamics, a key executioner in neurodegenerative diseases. Mitochondrion.

[11] Caspersen C (2005). Mitochondrial A β: A potential focal point for neuronal metabolic dysfunction in Alzheimer’s disease. Mitochondrion.

[12] Yao J (2009). Mitochondrial bioenergetic deficit precedes Alzheimer’s pathology in female mouse model of Alzheimer’s disease. Proc. Natl. Acad. Sci. USA..

[13] Parker WD, Parks JK, Swerdlow RH (2008). Complex I deficiency in Parkinson’s disease frontal cortex. Brain Res..

[14] Gunnewiek TMK (2020). Impairs human neuronal development and reduces neuronal network activity and synchronicity m 3243A > G-induced mitochondrial dysfunction impairs human neuronal development and reduces neuronal network activity and synchronicity. Brain Res..

[15] Sterky FH, Larsson NG (2008). Complex I: A complex gateway to the powerhouse. Cell Metab..

[16] Ingraham CA (2009). NDUFS4: Creation of a mouse model mimicking a complex I disorder. Mitochondrion.

[17] Kahlhöfer F, Kmita K, Wittig I, Zwicker K, Zickermann V (2017). Accessory subunit NUYM (NDUFS4) is required for stability of the electron input module and activity of mitochondrial complex I. Biochim. Biophys. Acta Bioenerg..

[18] Shrikhande DY, Kalakoti P, Syed MMA, Ahya K, Singh G (2010). A rare mitochondrial disorder: Leigh syndrome–a case report. Ital. J. Pediatr..

[19] Lamont RE (2017). A novel NDUFS4 frameshift mutation causes Leigh disease in the Hutterite population. Am. J. Med. Genet. A.

[20] Quintana A, Kruse SE, Kapur RP, Sanz E, Palmiter RD (2010). Complex I deficiency due to loss of Ndufs4 in the brain results in progressive encephalopathy resembling Leigh syndrome. Proc. Natl. Acad. Sci. USA..

[21] Bolea I (2019). Defined neuronal populations drive fatal phenotype in a mouse model of leigh syndrome. Elife.

[22] Chen B (2017). Loss of mitochondrial Ndufs4 in striatal medium spiny neurons mediates progressive motor impairment in a mouse model of leigh syndrome. Front. Mol. Neurosci..

[23] Choi WS (2017). Conditional deletion of Ndufs4 in dopaminergic neurons promotes Parkinson’s disease-like non-motor symptoms without loss of dopamine neurons. Sci. Rep..

[24] Kim HW (2015). Genetic reduction of mitochondrial complex I function does not lead to loss of dopamine neurons invivo. Neurobiol. Aging.

[25] Spillane M, Ketschek A, Merianda TT, Twiss JL, Gallo G (2013). Mitochondria coordinate sites of axon branching through localized intra-axonal protein synthesis. Cell Rep..

[26] Berkeley JL, Levey AI (2000). Muscarinic activation of mitogen-activated protein kinase in PC12 cells. J. Neurochem..

[27] Resende RR, Adhikari A (2009). Cholinergic receptor pathways involved in apoptosis, cell proliferation and neuronal differentiation. Cell Commun. Signal..

[28] VanDeMark KL, Guizzetti M, Giordano G, Costa LG (2009). The activation of M 1, muscarinic receptor signaling induces neuronal differentiation in pyramidal hippocampal neurons. J. Pharmacol. Exp. Ther..

[29] Cartoni R (2016). The mammalian-specific protein armcx1 regulates mitochondrial transport during axon regeneration. Neuron.

[30] Trigo D, Goncalves MB, Corcoran JPT (2019). The regulation of mitochondrial dynamics in neurite outgrowth by retinoic acid receptor β signaling. FASEB J..

[31] Cheng HC, Ulane CM, Burke RE (2010). Clinical progression in Parkinson disease and the neurobiology of axons. Ann. Neurol..

[32] Scheff SW, Price DA, Schmitt FA, Dekosky ST, Mufson EJ (2007). Synaptic alterations in CA1 in mild Alzheimer disease and mild cognitive impairment. Neurology.

[33] Kayser EB, Sedensky MM, Morgan PG (2016). Region-specific defects of respiratory capacities in the Ndufs4(KO) mouse brain. PLoS ONE.

[34] Tait SWG, Green DR (2012). Mitochondria and cell signalling. J. Cell Sci..

[35] Zhang WL (2018). Neuroprotective effect of tanshinone IIA weakens spastic cerebral palsy through inflammation, p38MAPK and VEGF in neonatal rats. Mol. Med. Rep..

[36] Gui C (2020). p38 MAPK-DRP1 signaling is involved in mitochondrial dysfunction and cell death in mutant A53T α-synuclein model of Parkinson’s disease. Toxicol. Appl. Pharmacol..

[37] Zhou J (2019). NADPH ameliorates MPTP-induced dopaminergic neurodegeneration through inhibiting p38MAPK activation. Acta Pharmacol. Sin..

[38] Pyakurel A, Savoia C, Hess D, Scorrano L (2015). Extracellular regulated kinase phosphorylates mitofusin 1 to control mitochondrial morphology and apoptosis. Mol. Cell.

[39] Newbern J, Taylor A, Robinson M, Lively MO, Milligan CE (2007). c-Jun N-terminal kinase signaling regulates events associated with both health and degeneration in motoneurons. Neuroscience.

[40] Ohta KI (2017). Prolonged maternal separation attenuates BDNF-ERK signaling correlated with spine formation in the hippocampus during early brain development. J. Neurochem..

[41] Kanterewicz BI (2000). The extracellular signal-regulated kinase cascade is required for NMDA receptor-independent LTP in area CA1 but not area CA3 of the hippocampus. J. Neurosci..

[42] Kushner SA (2005). Modulation of presynaptic plasticity and learning by the H-ras/extracellular signal-regulated kinase/synapsin I signaling pathway. J. Neurosci..

[43] Vara H, Onofri F, Benfenati F, Sassoè-Pognetto M, Giustetto M (2009). ERK activation in axonal varicosities modulates presynaptic plasticity in the CA3 region of the hippocampus through synapsin I. Proc. Natl. Acad. Sci. USA..

[44] Alonso M, Medina JH, Pozzo-Miller L (2004). ERK1/2 activation is necessary for BDNF to increase dendritic spine density in hippocampal CA1 pyramidal neurons. Learn. Mem..

[45] Brun A, Liu X, Erikson C (1995). Synapse loss and gliosis in the molecular layer of the cerebral cortex in Alzheimer’s disease and in frontal lobe degeneration. Neurodegeneration.

[46] Ekmark-Lewén S (2013). Traumatic axonal injury in the mouse is accompanied by a dynamic inflammatory response, astroglial reactivity and complex behavioral changes. J. Neuroinflammation.

[47] Chaudhari N, Ravanan P (2018). Bardoxolone methyl induces neuritogenesis in Neuro2a cells. Pharmacol. Rep..

[48] Kagawa Y (2015). Fatty acid-binding protein 7 regulates function of caveolae in astrocytes through expression of caveolin-1. Glia.

[49] Frezza C, Cipolat S, Scorrano L (2007). Organelle isolation: Functional mitochondria from mouse liver, muscle and cultured filroblasts. Nat. Protoc..

[50] Kagawa Y (2020). Mitochondrial dysfunction in GnRH neurons impaired GnRH production. Biochem. Biophys. Res. Commun..

[51] Chen G (2009). Cyanidin-3-glucoside reverses ethanol-induced inhibition of neurite outgrowth: Role of glycogen synthase kinase 3 beta. Neurotox. Res..

