# Supplementary material for: Ndufs4 ablation decreases synaptophysin expression in hippocampus
Source: Sci Rep. 2021 May 26;11:10969. doi: 10.1038/s41598-021-90127-4 (PMC8155116; doi:10.1038/s41598-021-90127-4)
Supplement: Supplementary file 1 — Supplementary Information. [file 41598_2021_90127_MOESM1_ESM.pdf]

Supplemental Information

Title

Ndufs4 ablation decreases synaptophysin expression in hippocampus

Authors

Subrata Kumar Shil[1], Yoshiteru Kagawa[1], Banlanjo Abdulaziz Umaru[1], Fumika Nanto-Hara[2], Hirofumi Miyazaki[1], Yui Yamamoto[3], Shuhei Kobayashi[1], Chitose Suzuki[4], Takaaki Abe[4] & Yuji Owada[1]

Institutional affiliations

[1]Department of Organ Anatomy, Tohoku University Graduate School of Medicine, Sendai, 980-8575, Japan, [2]Division of Animal Metabolism and Nutrition, Institute of Livestock and Grassland Science, National Agriculture and Food Research Organization, Tsukuba 305-0901, Japan, [3]Department of Anatomy, Tohoku Medical and Pharmaceutical University, Sendai, 981-0905, Japan. [4]Department of Nephrology, Endocrinology and Vascular Medicine, Tohoku University Graduate School of Medicine, 980-8575, Japan.

Correspondence Author

Yoshiteru Kagawa and Yuji Owada, Department of Organ Anatomy, Tohoku University Graduate School of Medicine, 2-1 Seiryo-machi, Aoba-ku, Sendai, Miyagi. 980-8575 Japan, Tel: +81-22-717-7600; Fax: +81-22-717-8041. E-mail: kagawa@med.tohoku.ac.jp, owada@med.tohoku.ac.jp

Supplemental Figure 1

A

		ct mean ± SD	RQ mean
Heart	WT	21.1585 ± 3.3890	4.1982
	KO	34.8733 ± 0.0001	0.0001
Cerebral cortex	WT	23.0676 ± 0.9038	1.0000
	KO	32.8363 ± 0.0003	0.0003
Hippocampus	WT	22.6872 ± 1.4800	1.4581
	KO	35.0271 ± 0.0005	0.0002
Cerebellum	WT	21.9253 ± 1.0096	1.3824
	KO	35.1855 ± 0.0001	0.0001

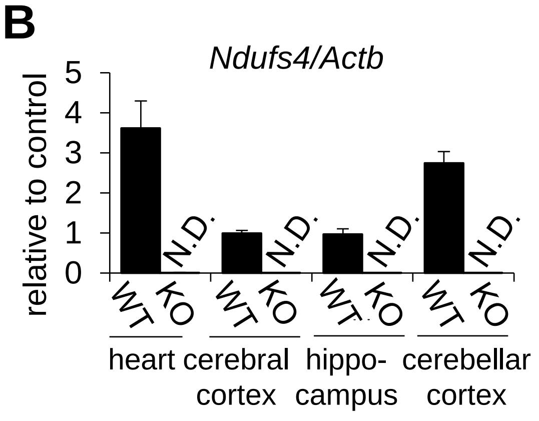

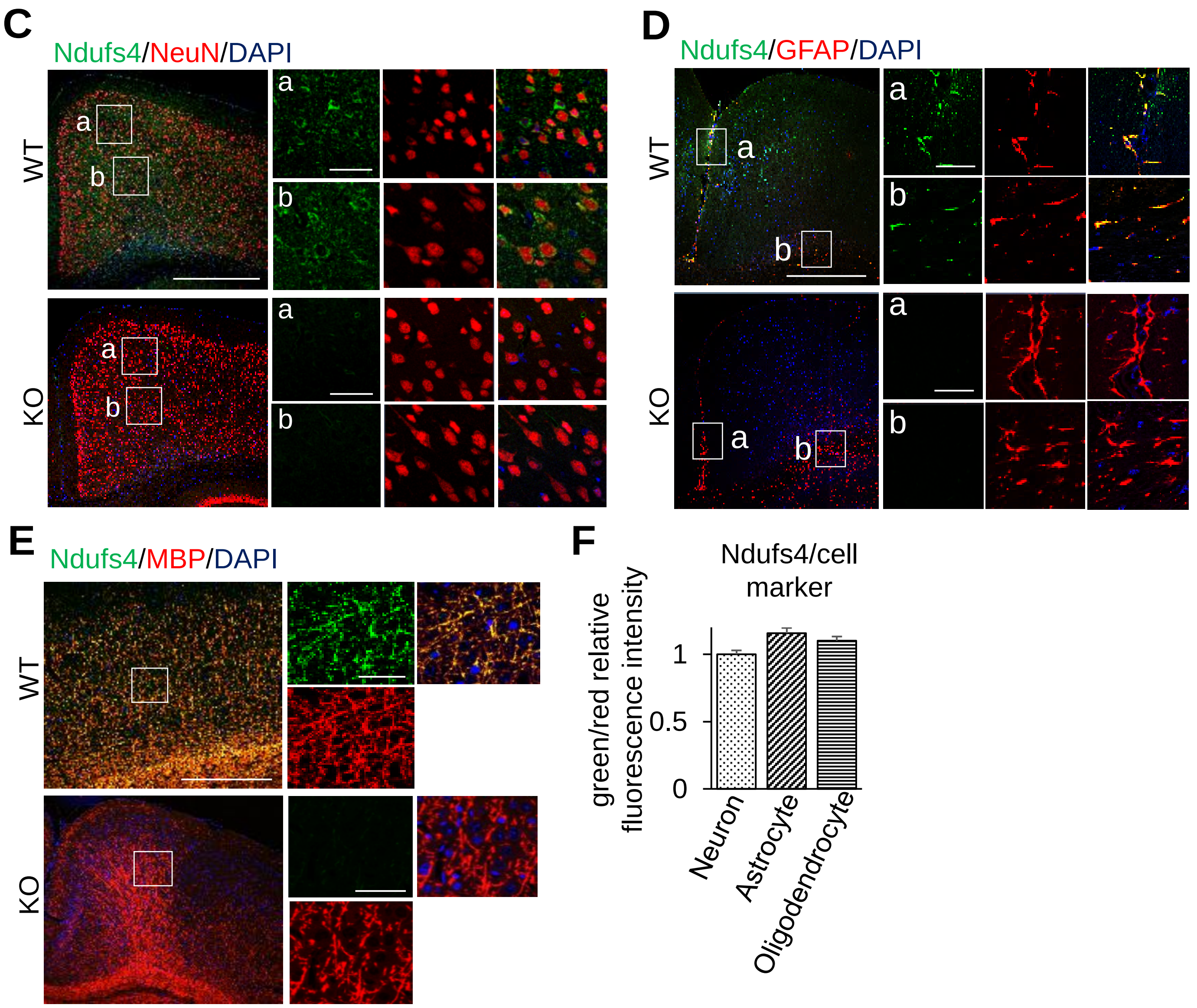

Supplemental Figure 1. (A, B) qPCR for *Ndufs4* expression in heart and different parts of brain of WT (n=6) and Ndufs4-KO (n=6) mouse. (C, D, E) Co-immuofluorescence staining of Ndufs4 (Green) with NeuN (C), GFAP (D) and MBP (E) (red) in cerebral cortex of WT (n=6) and Ndufs4-KO (n=6) mouse. Boxed area is enlarged on right. Scale for low magnification: 500 µm, for high magnification: 40 µm. (F, G) The fluorescence intensity quantification of Ndufs4 immunoreactivity using ImageJ (NIH, USA). Error bars denote standard error of mean.

Supplemental Figure 2

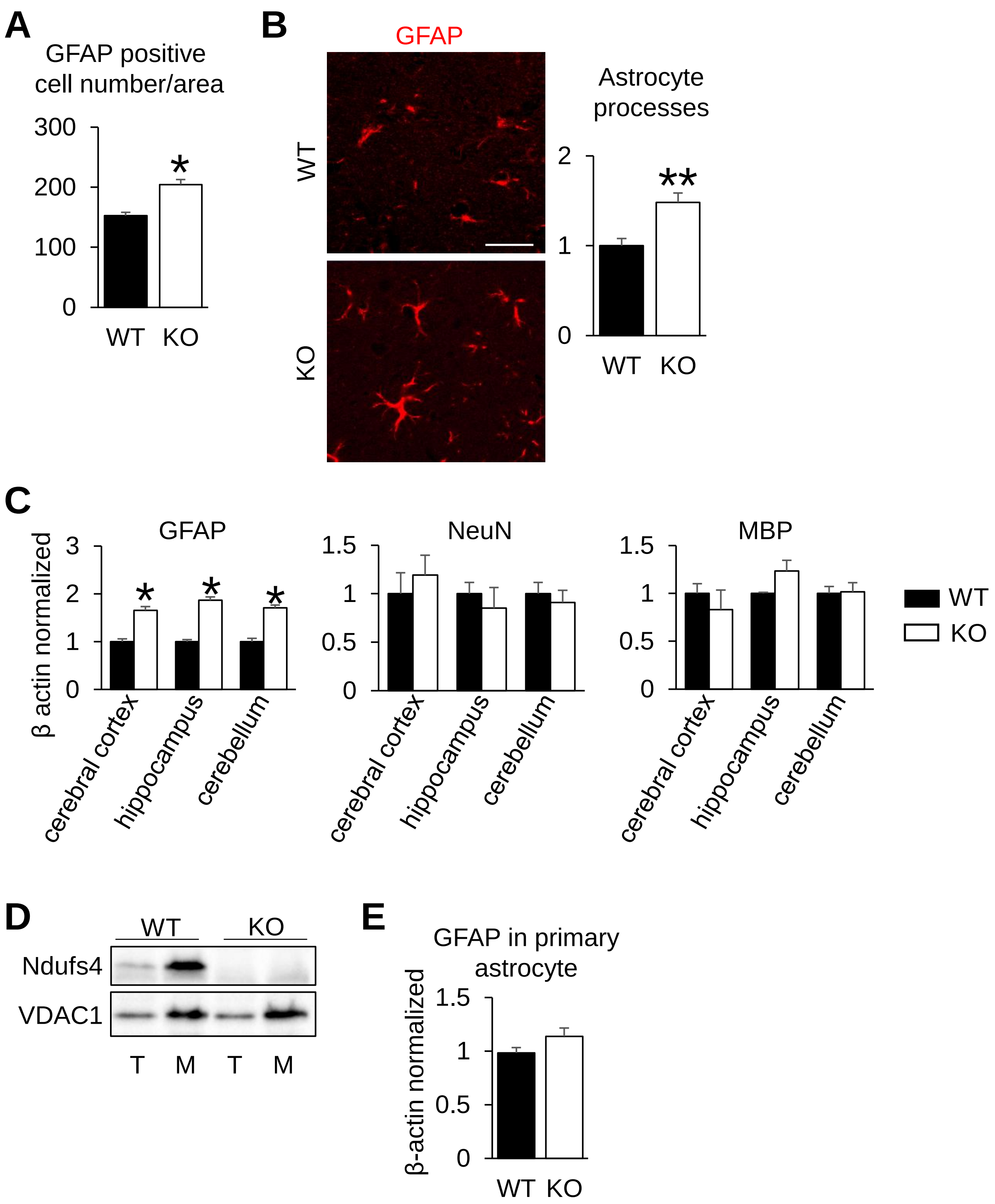

Supplemental figure 2 (A, B) Counting of GFAP positive cells and their processes in hippocampus of WT (n=6) and Ndufs4-KO (n=6) mouse by ImageJ. Scale bar: 20 μm. (C) Analysis of band density using ImageJ of GFAP, NeuN and MBP protein in hippocampus of WT (n=6) and Ndufs4-KO (n=6) mouse. (D) Representative Western blot images of Ndufs4 protein expression in WT and KO primary astrocytes. T: total protein, M: protein from isolated mitochondria. (E) Analysis of band density of GFAP protein in WT (n=3) and Ndufs4-KO (n=3) primary astrocytes using ImageJ.

Supplemental Figure 3

A

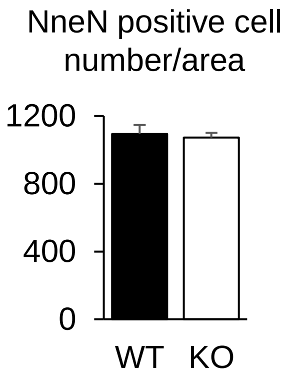

Supplemental figure 3. Counting of NeuN positive neuron number in hippocampus of WT (n=6) and Ndufs4-KO (n=6) mouse by ImageJ.

Supplemental Figure 4

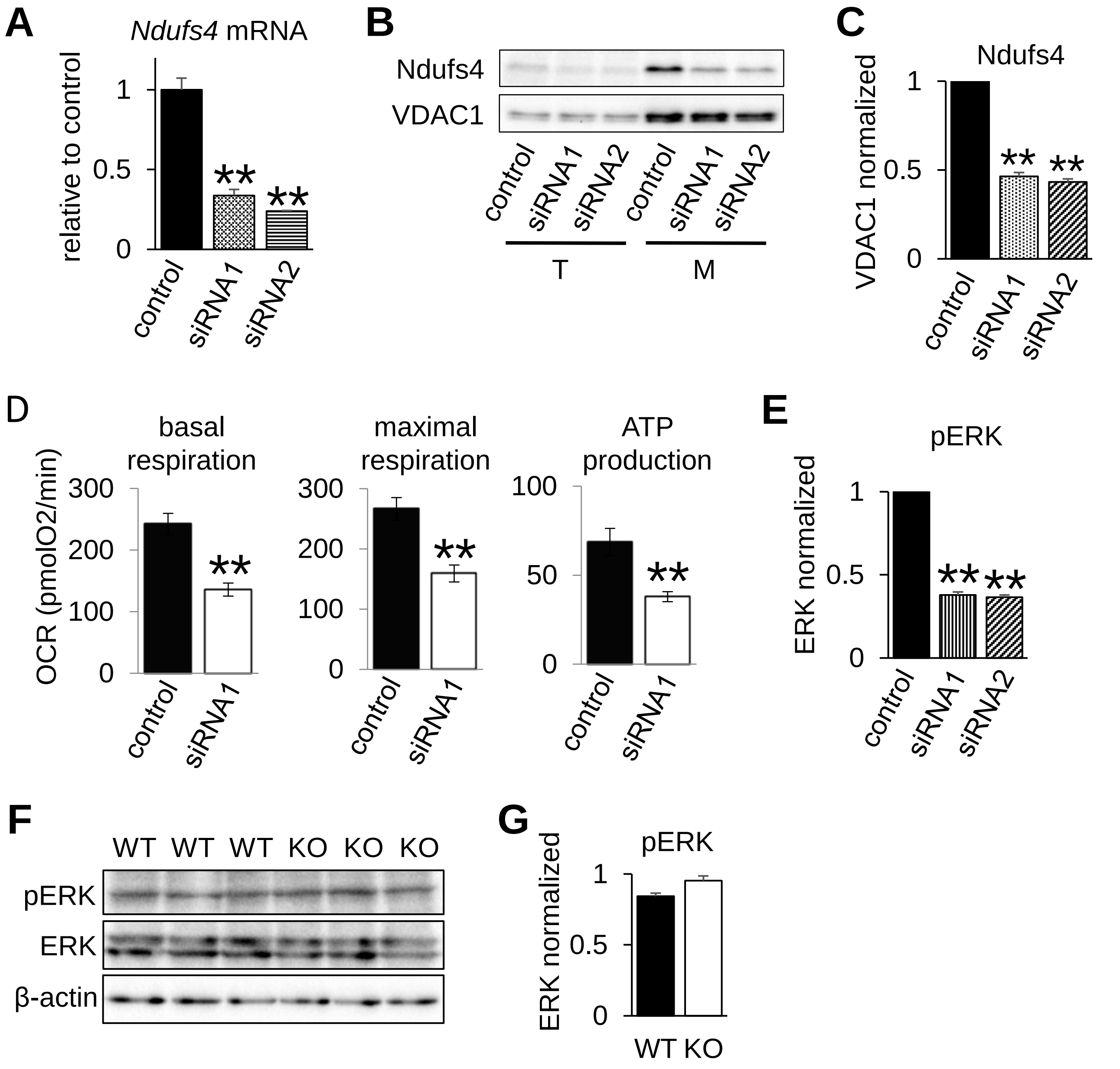

Supplemental figure 4 (A) Representative qPCR data for *Ndufs4* expression in control and Ndufs4 silenced Neuro-2a cells. Expression was normalized by *Actb*. (B) Representative images of Western blot analysis for Ndufs4 expression in control and Ndufs4 silenced Neuro-2a cells. T: total protein, M: protein from isolated mitochondria (C) Analysis of band density of Ndufs4 and pERK protein using ImageJ (n=3) (D) Mitochondrial respiration assay using XF24 analyser from control and Ndufs4 silenced Neuro-2a cells (n=3) (E) Analysis of band density of pERK protein using ImageJ (n=3) (F) Representative images of Western blot analysis of pERK and ERK protein expression in WT and KO hippocampus. (G) Band density analysis of pERK normalized to ERK expression in WT (n=6) and KO (n=6) hippocampus.

Supplemental Figure 5

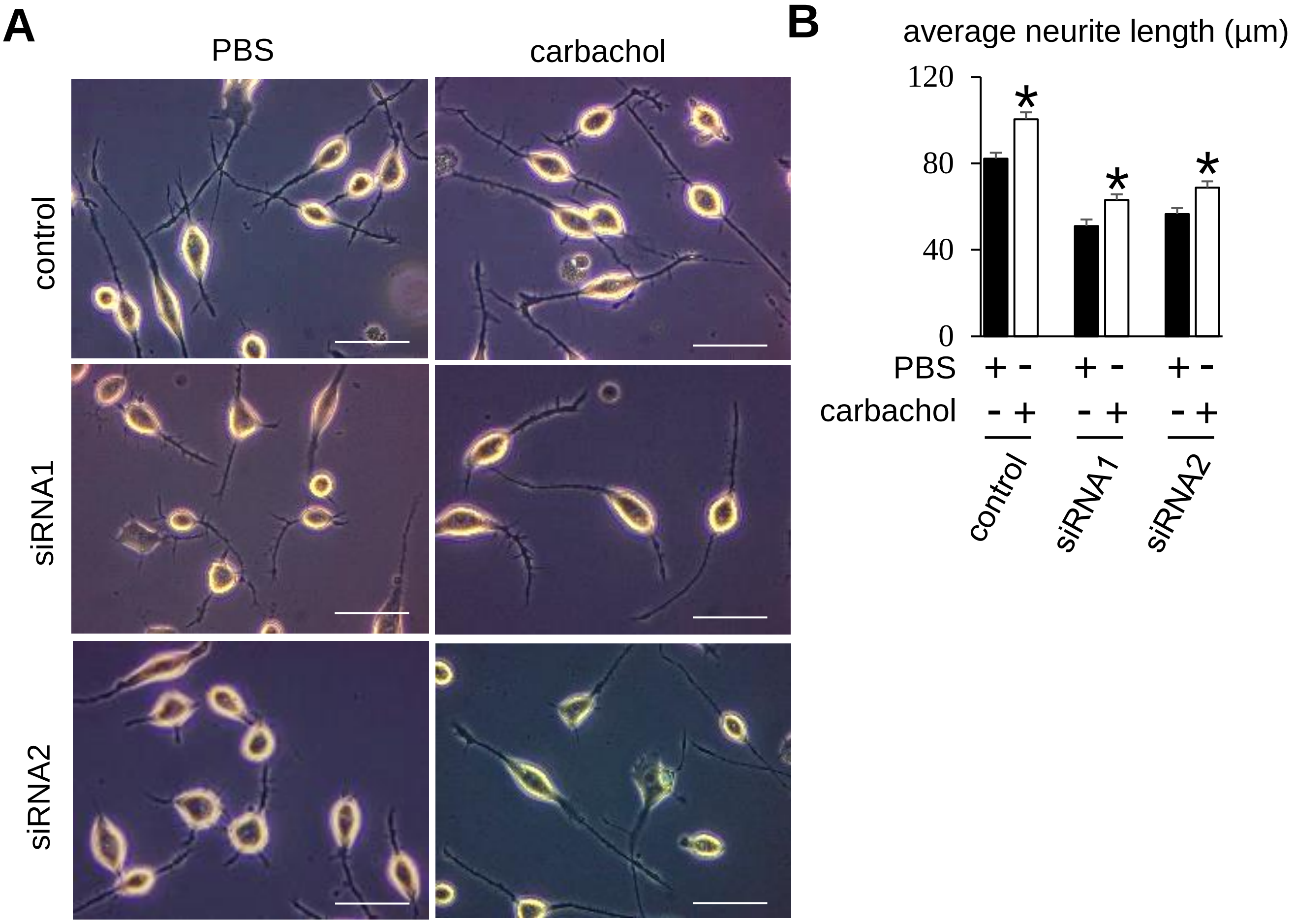

Supplemental figure 5 (A) Representative phase contrast image of neurite outgrowth in carbachol treated Ndufs4 silenced differentiated Neuro-2a cells. Scale bar 50 μm. (B) ImageJ analysis of average neurite length of differentiated cells (n=3).

Supplemental Figure 5

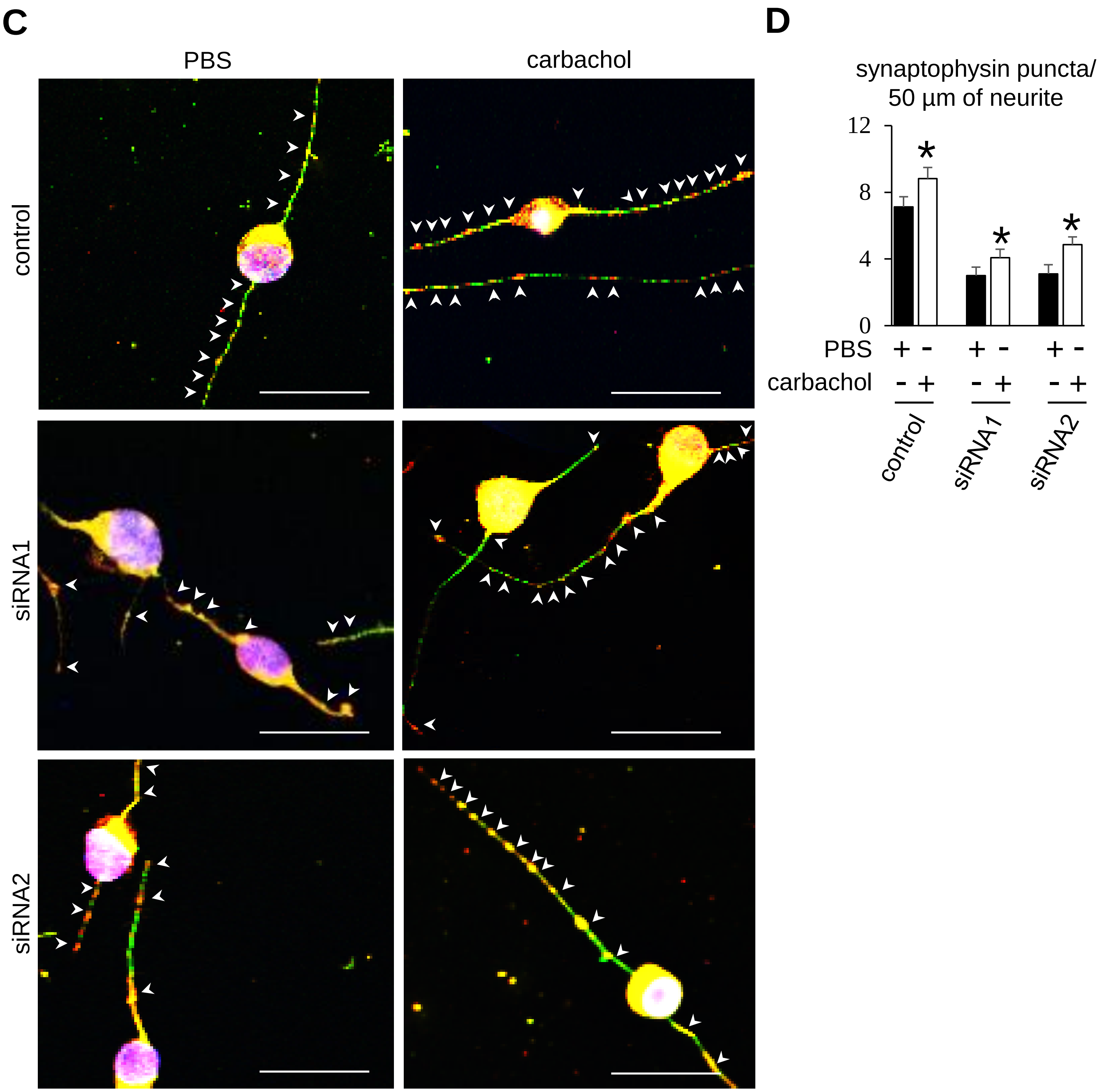

Supplemental figure 5 (C) Representative image of carbachol treated Neuro-2a cells immunostained for β-III tubulin and synaptophysin. Arrow indicates location of yellow coloured synaptophysin puncta in neurites. Scale bar 40 µm (D) ImageJ analysis of average number of synaptophysin puncta in neurites/50 µm (n=3).

The original gels/blots images

Red marked area was cropped to prepare the main figure. Separate bot with same sample was used for the β actin as endogenous control.

Figure 1 A for Ndufs4 (18 kDa)

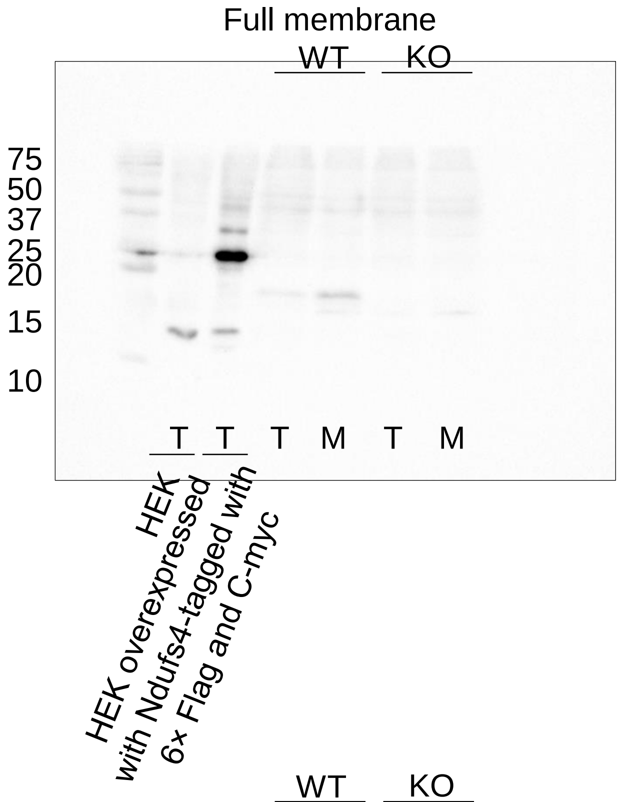

Exposure time 30 sec

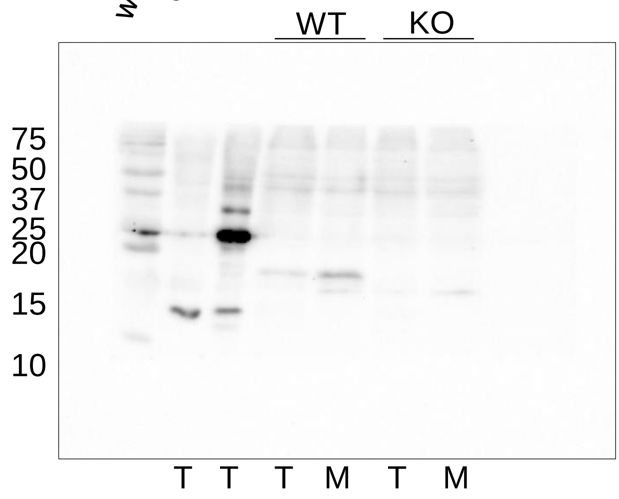

Exposure time 60 sec

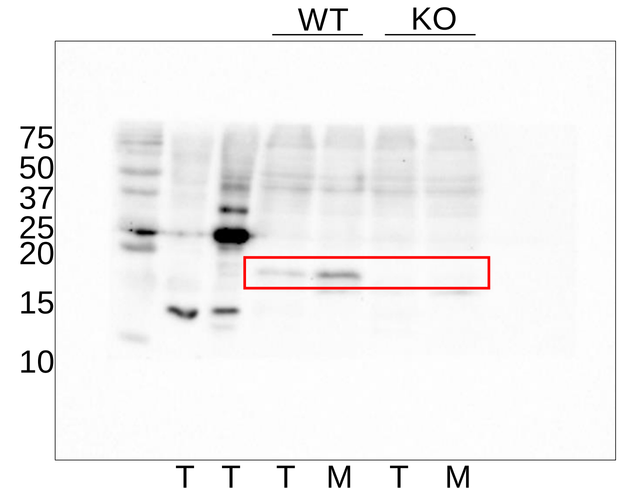

Exposure time 90 sec

The original gels/blots images

Figure 1 A for VDAC1 (31 kDa)

Full membrane

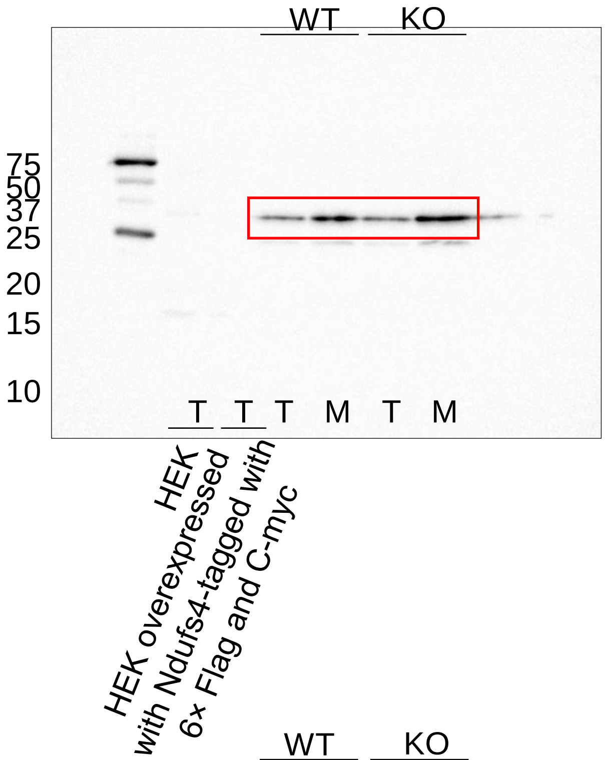

Exposure time 5 sec

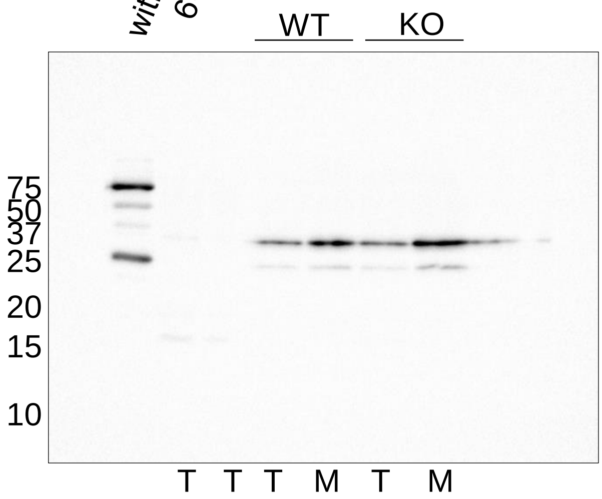

Exposure time 10 sec

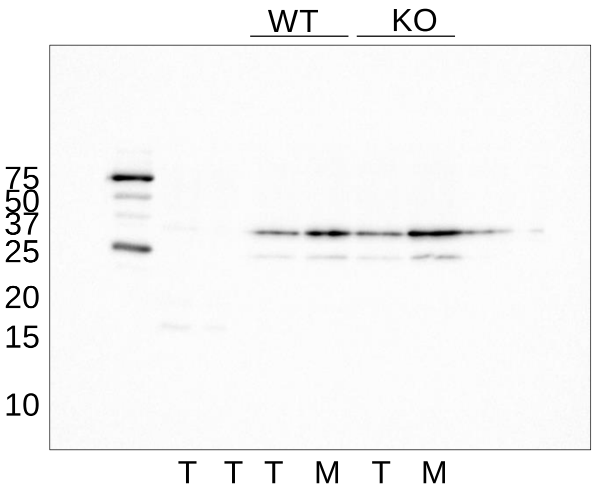

Exposure time 15 sec

The original gels/blots images

Figure 2D for GFAP (50 kDa)

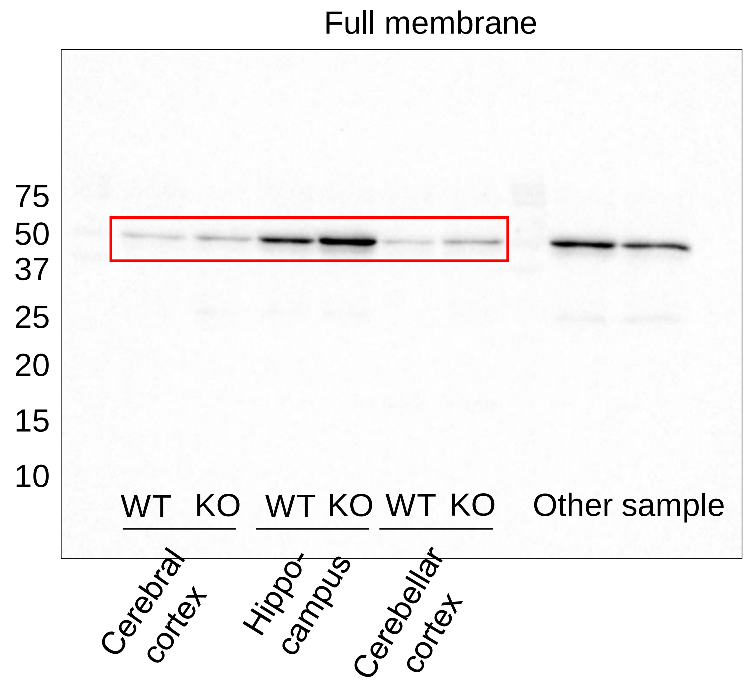

Exposure time 10 sec

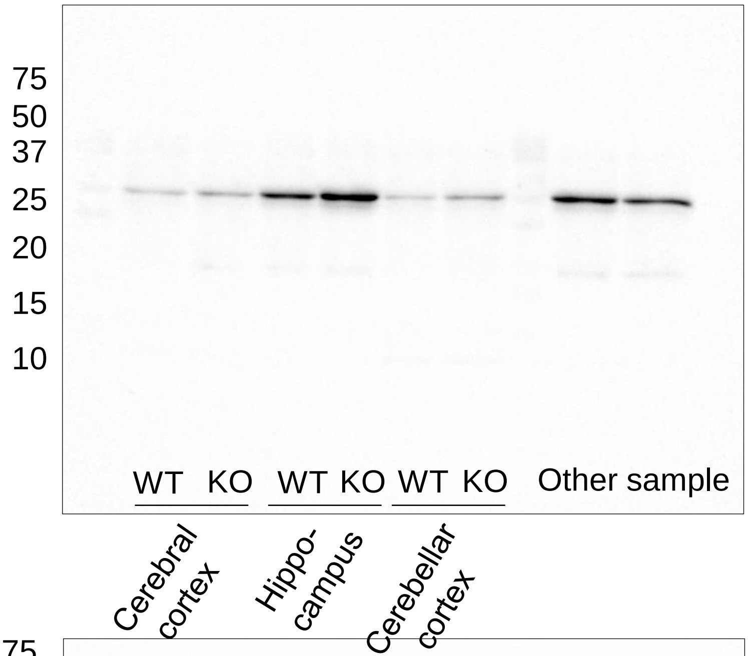

Exposure time 15 sec

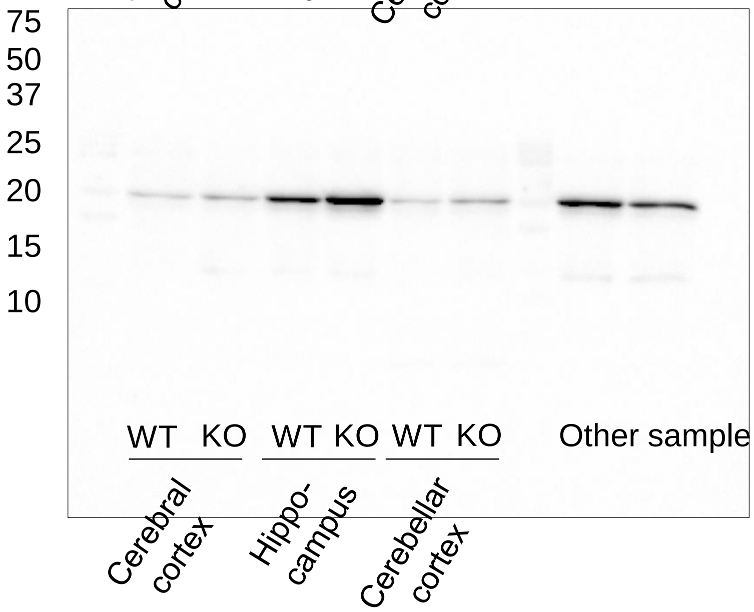

Exposure time 20 sec

The original gels/blots images

Figure 2D for NeuN 48 and 46 kDa

Full membrane

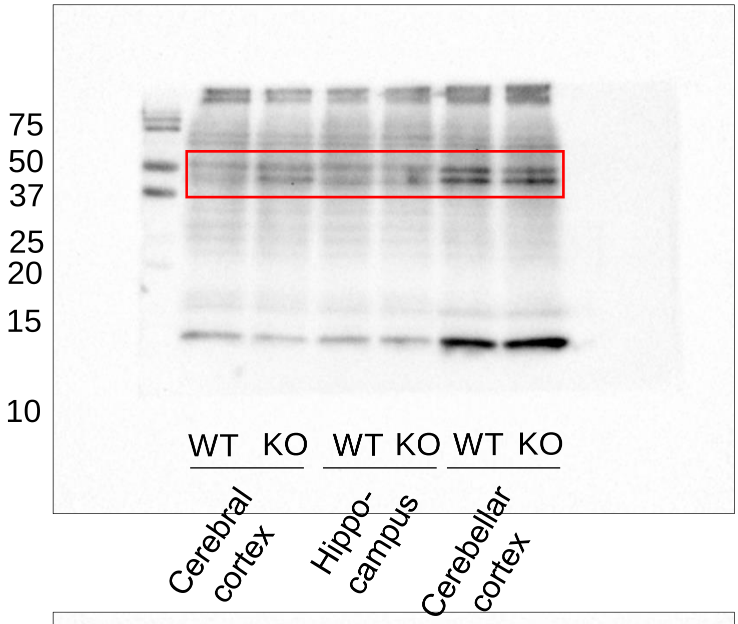

Exposure time 60 sec

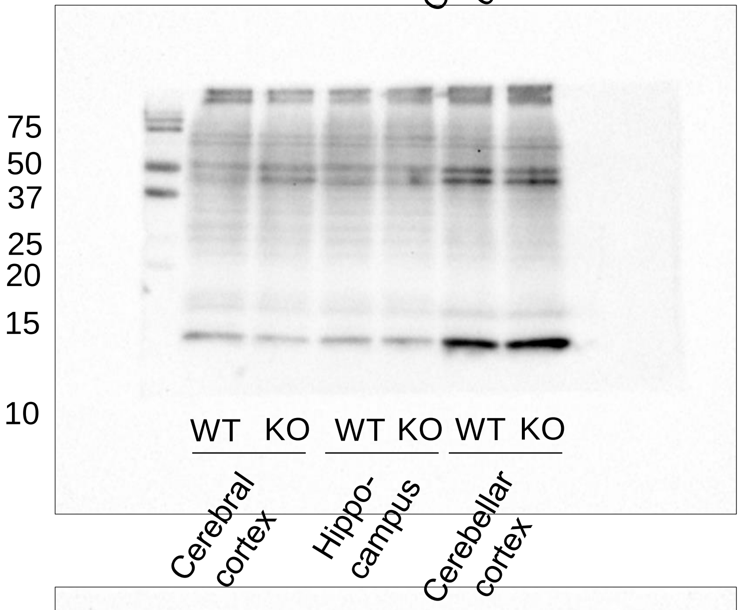

Exposure time 90 sec

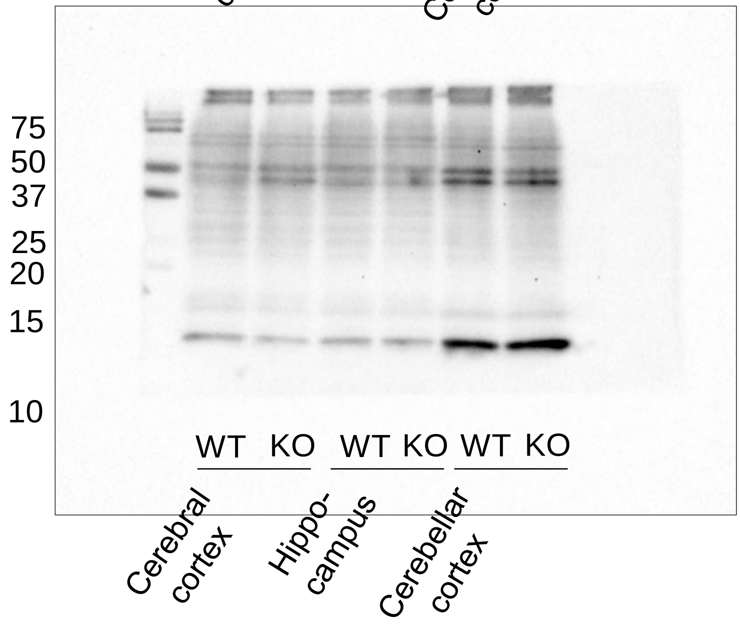

Exposure time 120 sec

The original gels/blots images

Figure 2D for MBP 22, 18 and 16 kDa

Full membrane

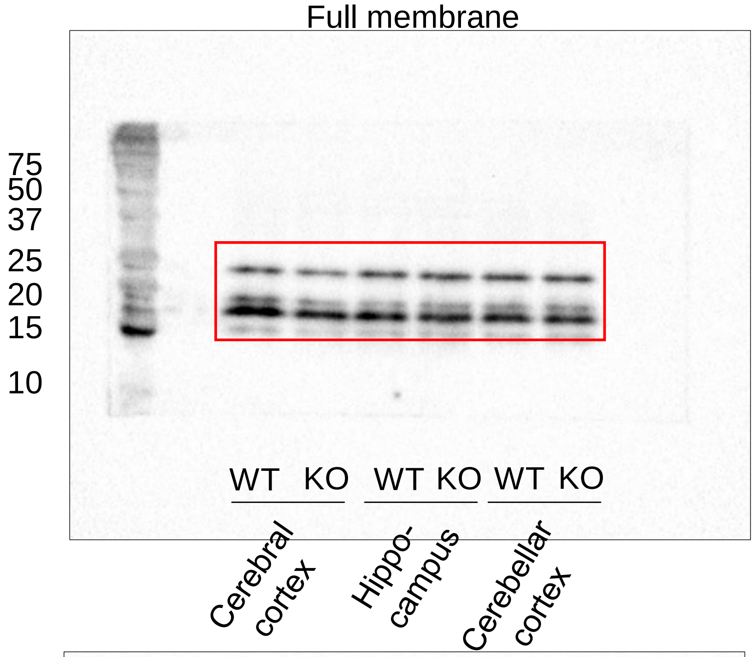

Exposure time 10 sec

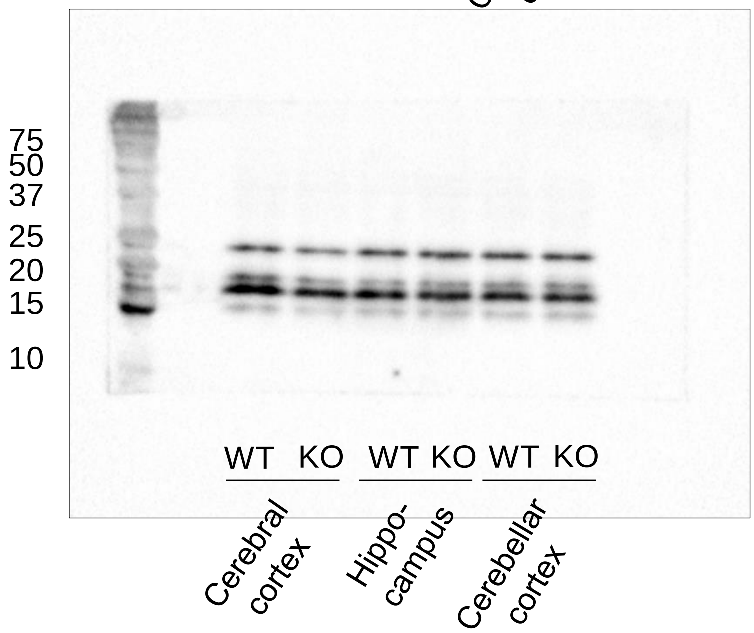

Exposure time 20 sec

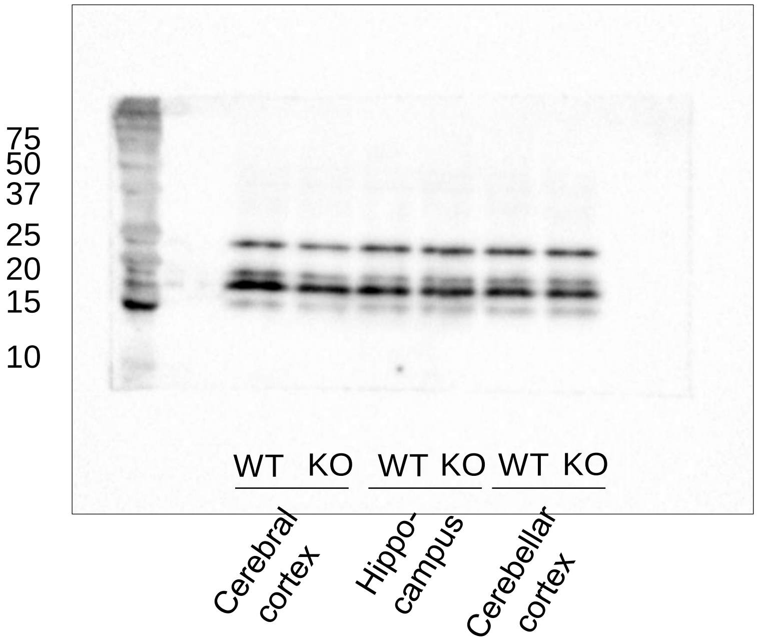

Exposure time 30 sec

The original gels/blots images
Figure 2D for β-actin (42 kDa)

Full membrane

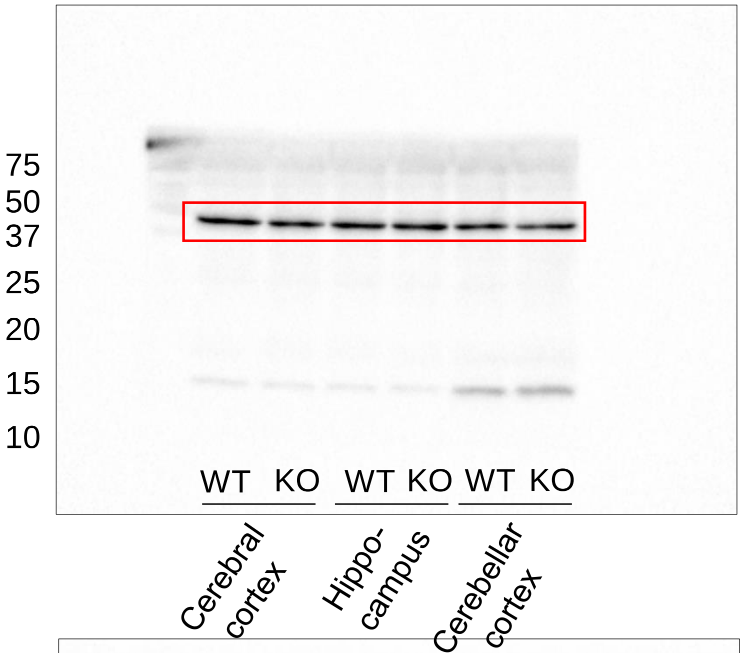

Exposure time 5 sec

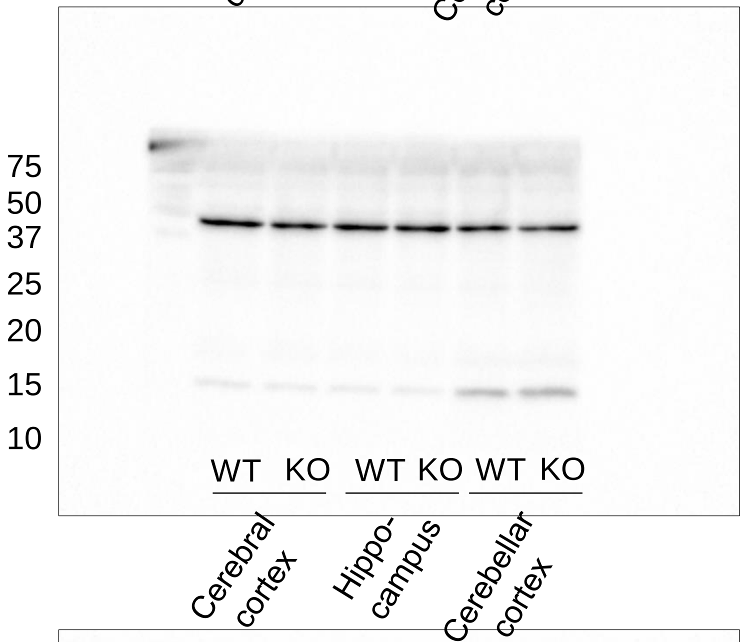

Exposure time 10 sec

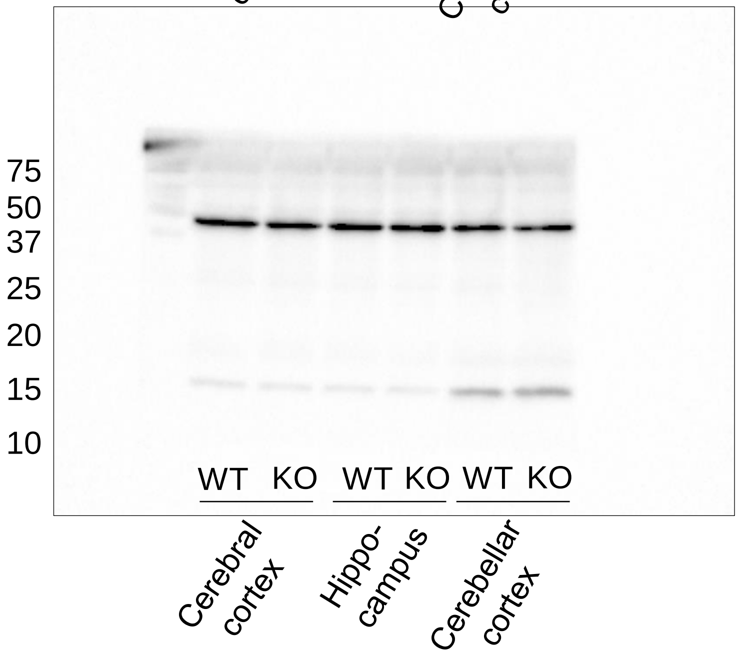

Exposure time 15 sec

The original gels/blots images

Figure 2E for GFAP (50 kDa)

Full membrane

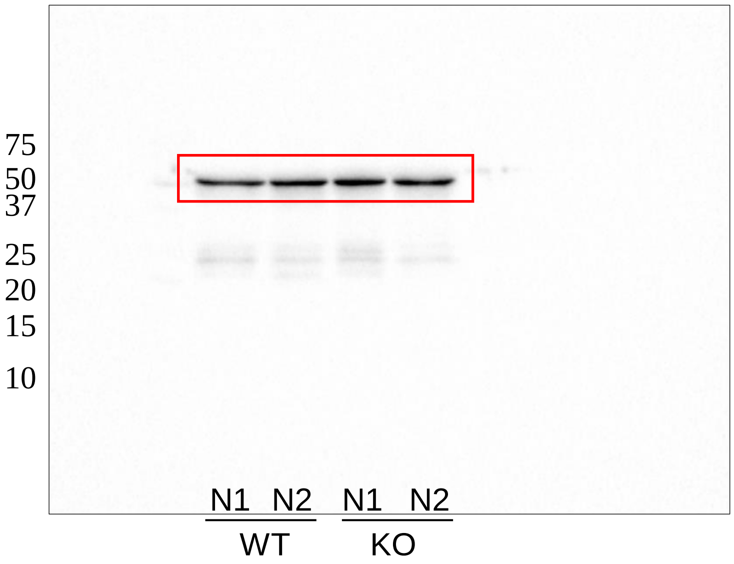

Exposure time 5 sec

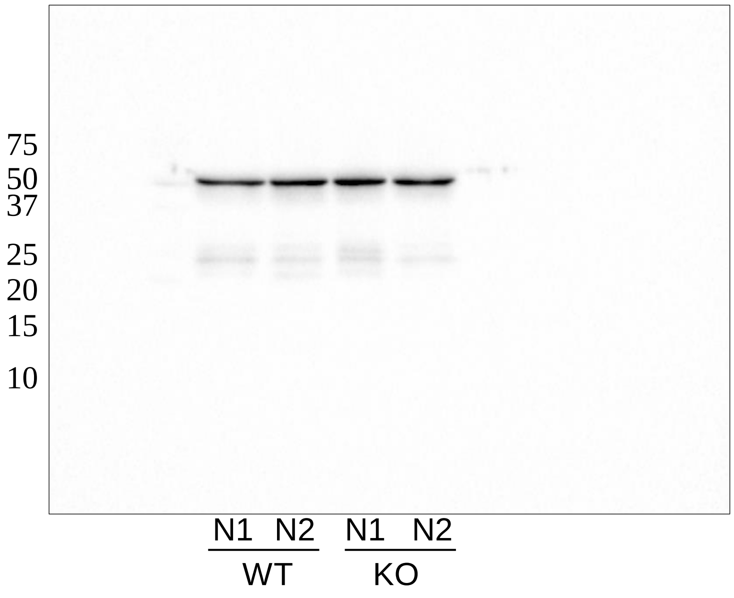

Exposure time 10 sec

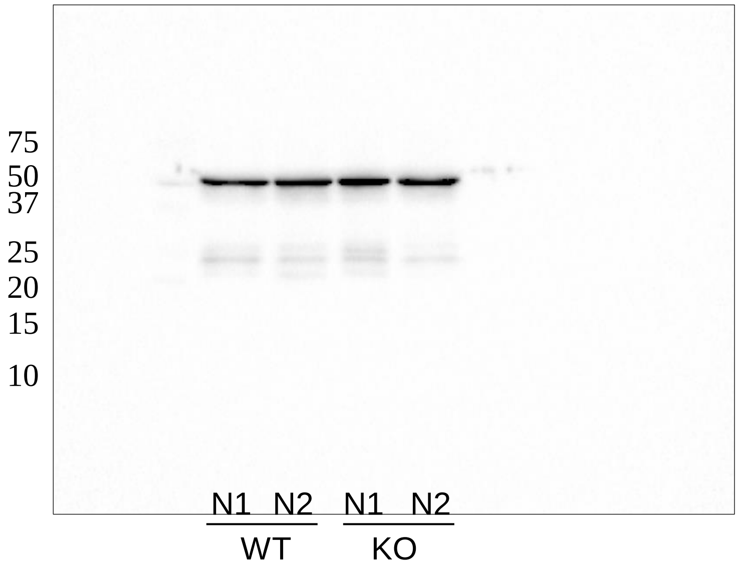

Exposure time 15 sec

The original gels/blots images
Figure 2E for β-actin (42 kDa)

Full membrane

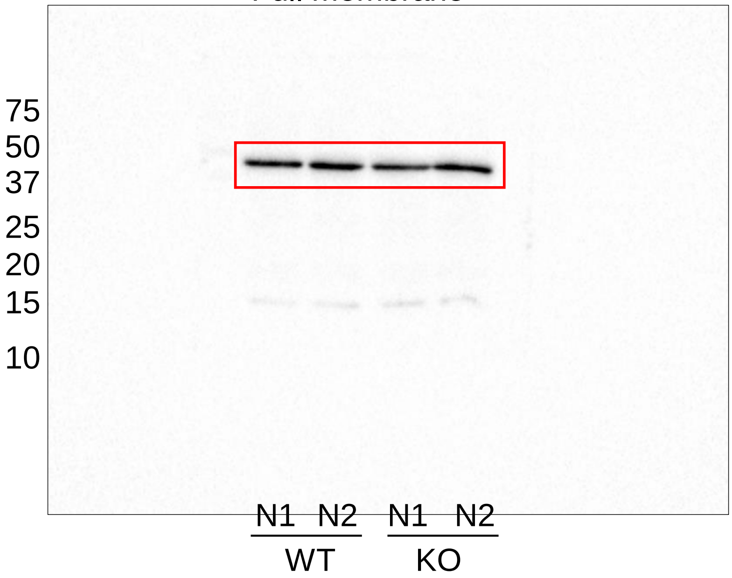

Exposure time 2 sec

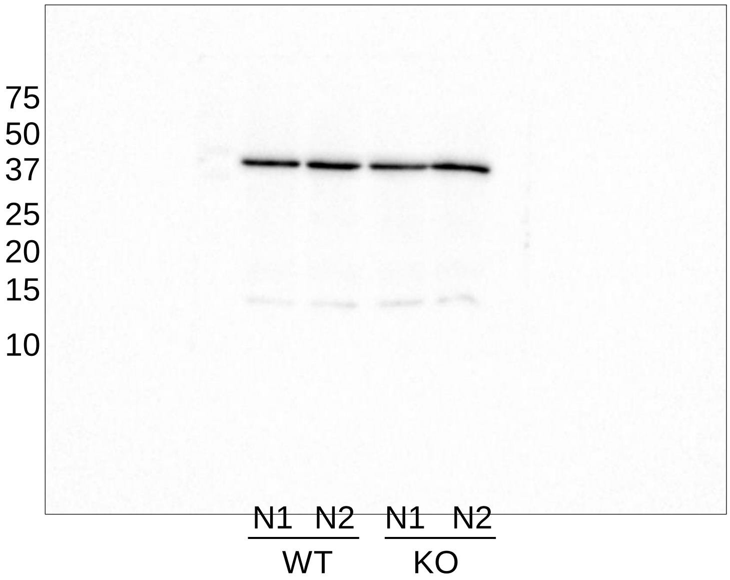

Exposure time 4 sec

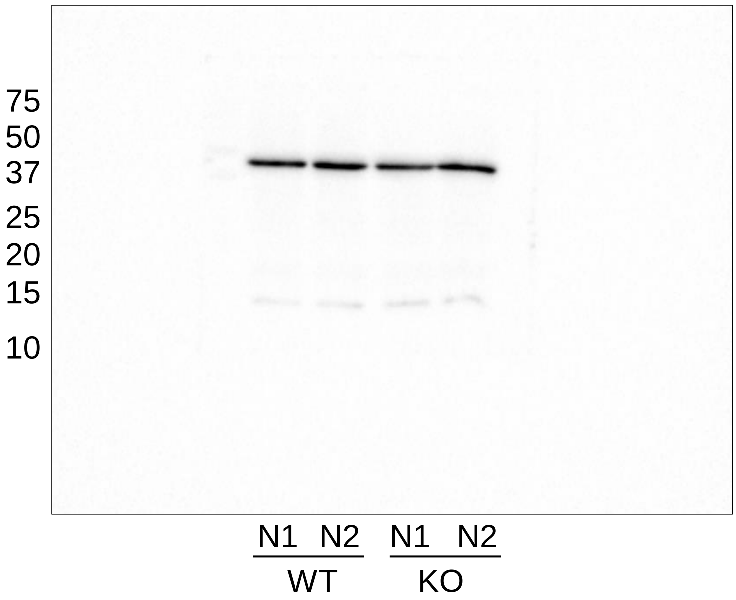

Exposure time 6 sec

The original gels/blots images

Figure 3B for synaptophysin (42 kDa)

Full membrane

75
50
37
25
20
15
10

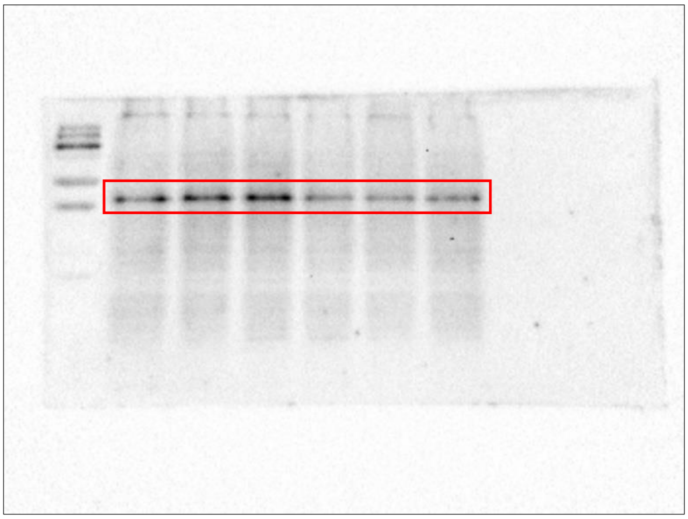

Exposure time 30 sec

WT WT WT KO KO KO

75
50
37
25
20
15
10

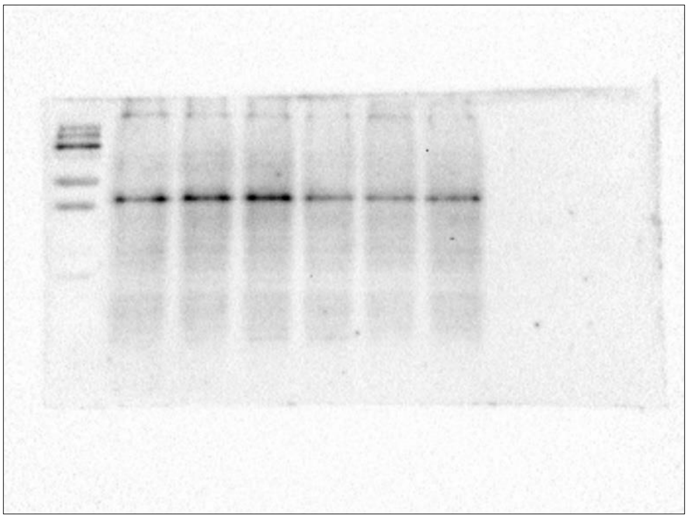

Exposure time 40 sec

WT WT WT KO KO KO

75
50
37
25
20
15
10

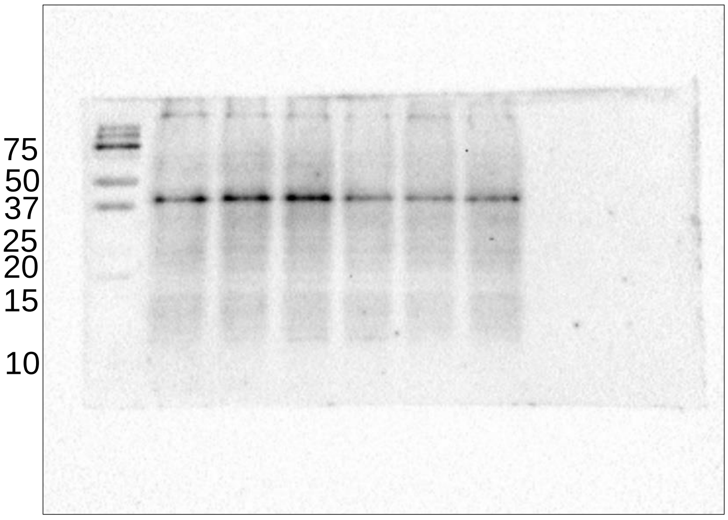

Exposure time 50 sec

WT WT WT KO KO KO

The original gels/blots images

Figure 3B for PSD95 (95 kDa)

Full membrane

250
150
100
75
50
37
25

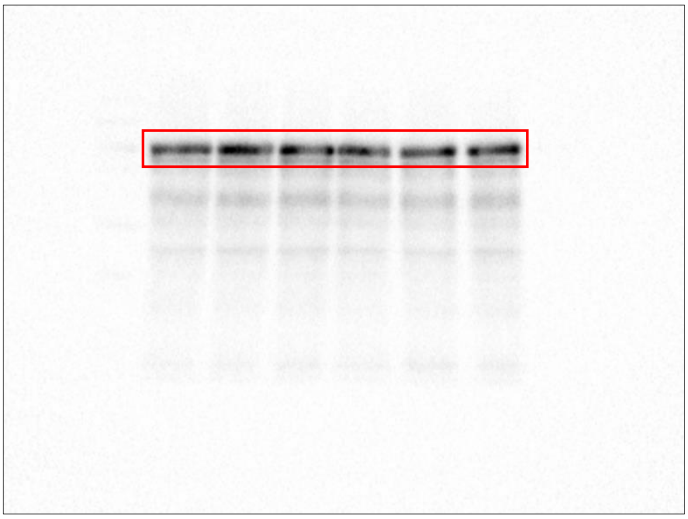

WT WT WT KO KO KO

Exposure time 1 sec

250
150
100
75
50
37
25

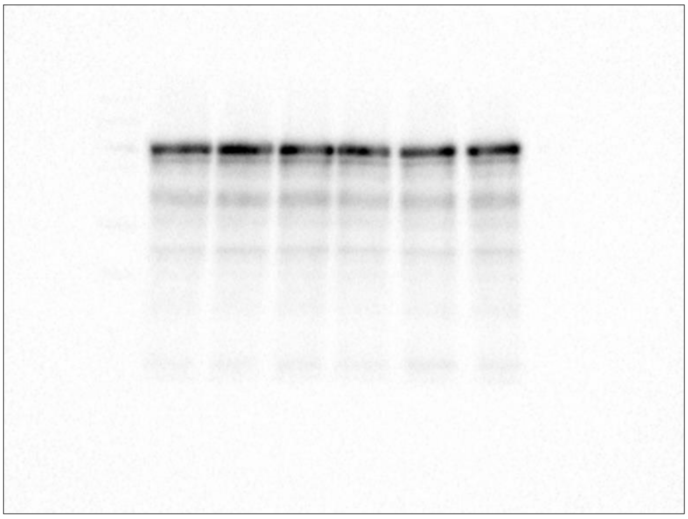

WT WT WT KO KO KO

Exposure time 2 sec

250
150
100
75
50
37
25

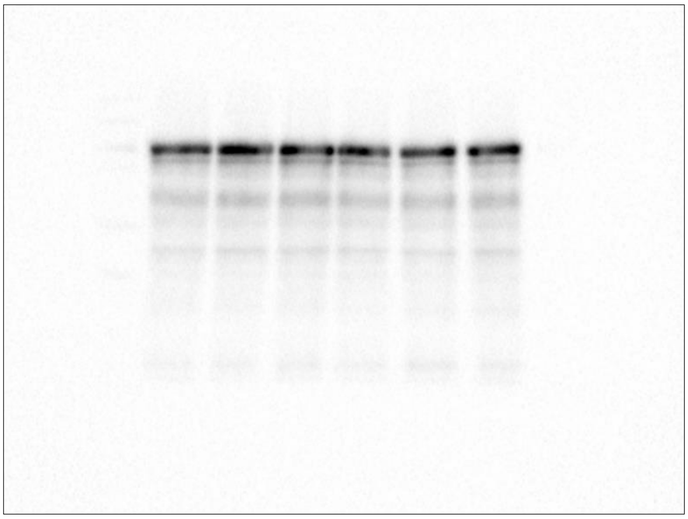

WT WT WT KO KO KO

Exposure time 3 sec

The original gels/blots images

Figure 3B for Vglut1 (61 kDa)

Full membrane

250
150
100
75
50
37
25

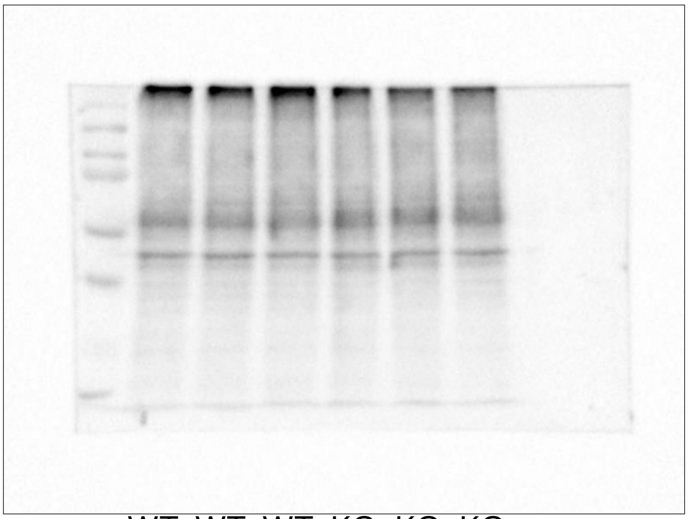

WT WT WT KO KO KO

Exposure time 2 sec

250
150
100
75
50
37
25

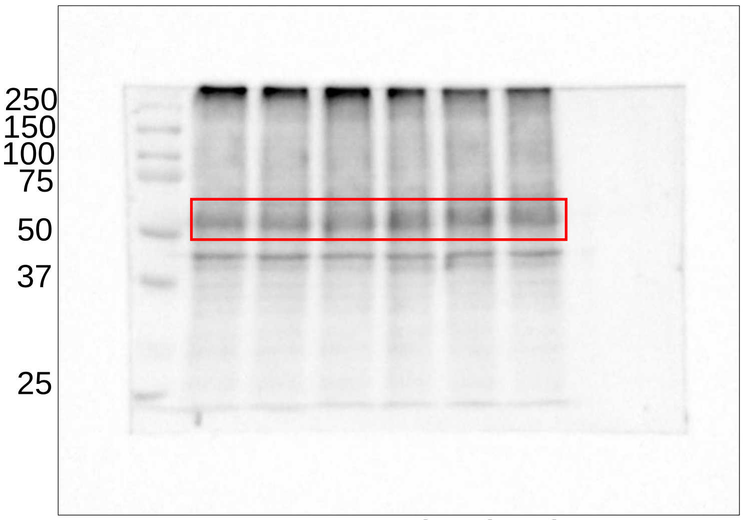

WT WT WT KO KO KO

Exposure time 4 sec

250
150
100
75
50
37
25

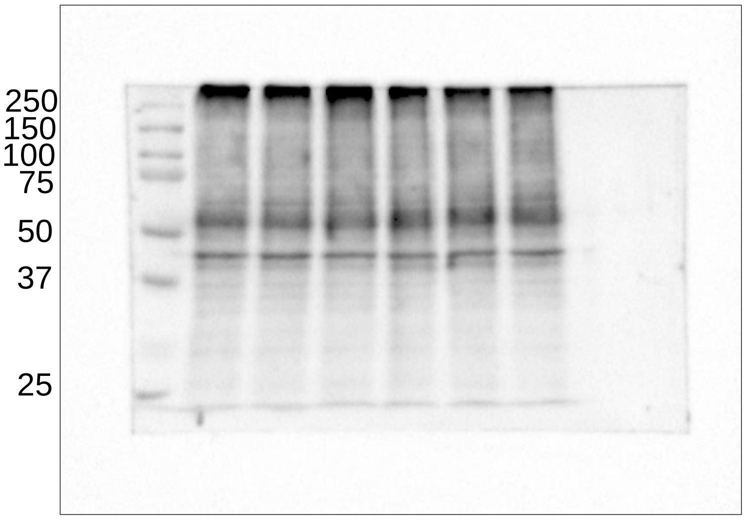

WT WT WT KO KO KO

Exposure time 4 sec

The original gels/blots images

Figure 3B for EAAT1 (60 kDa)

Full membrane

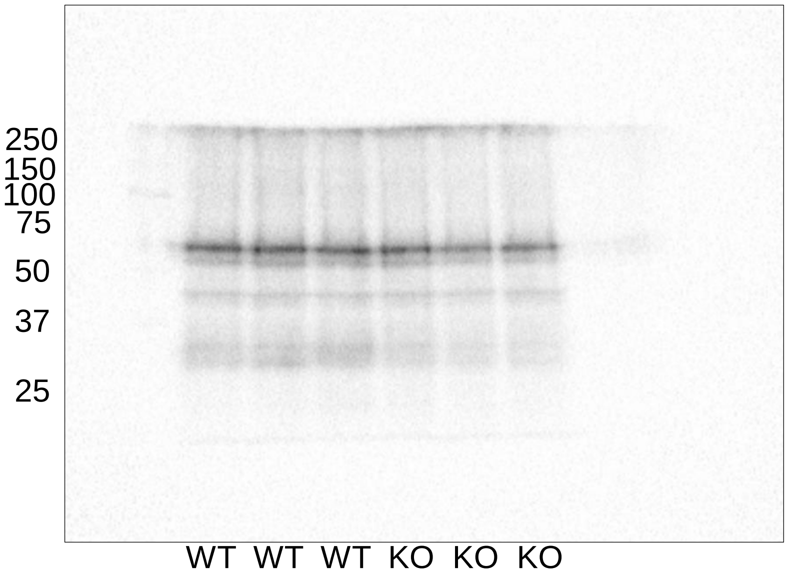

Exposure time 1 sec

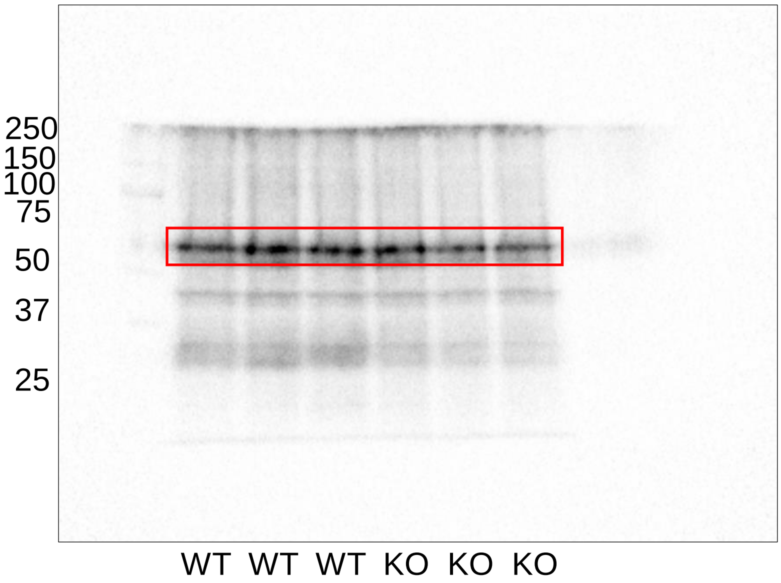

Exposure time 2 sec

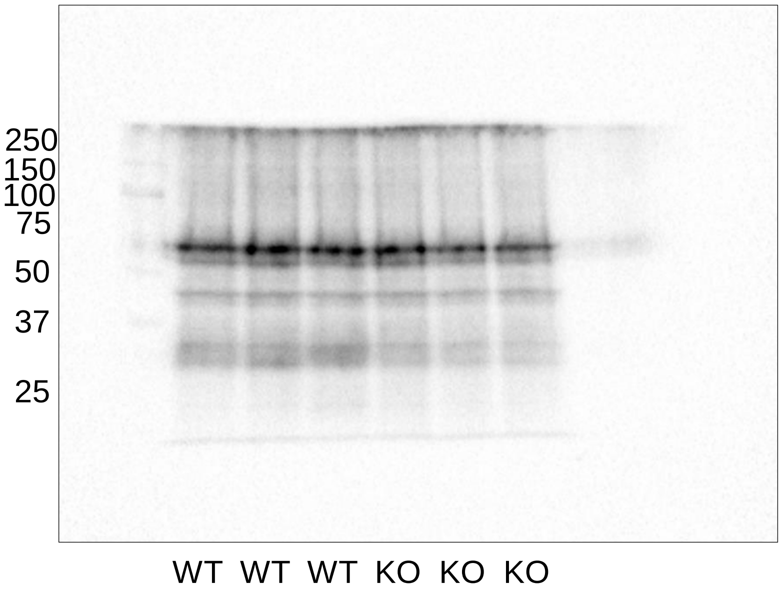

Exposure time 4 sec

The original gels/blots images

Figure 3B for EAAT2 (62 kDa)

Full membrane

250
150
100
75
50
37
25

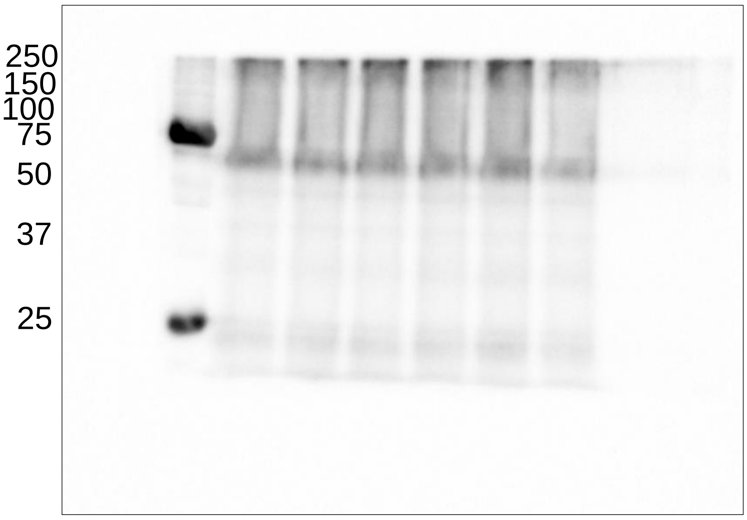

Exposure time 1 sec

WT WT WT KO KO KO

250
150
100
75
50
37
25

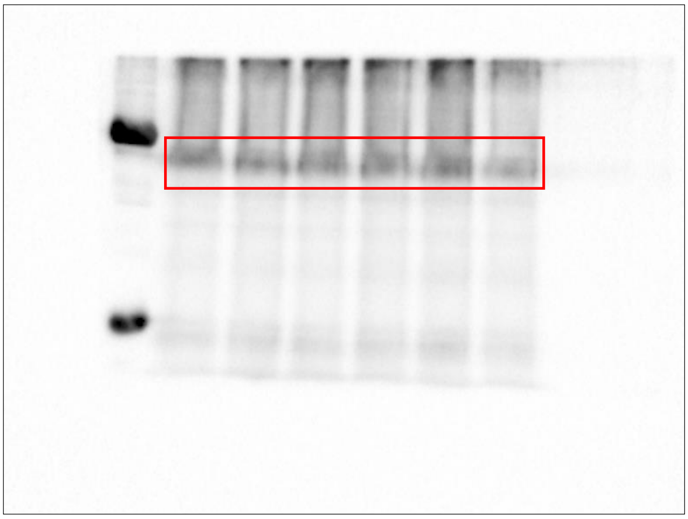

Exposure time 1.4 sec

WT WT WT KO KO KO

250
150
100
75
50
37
25

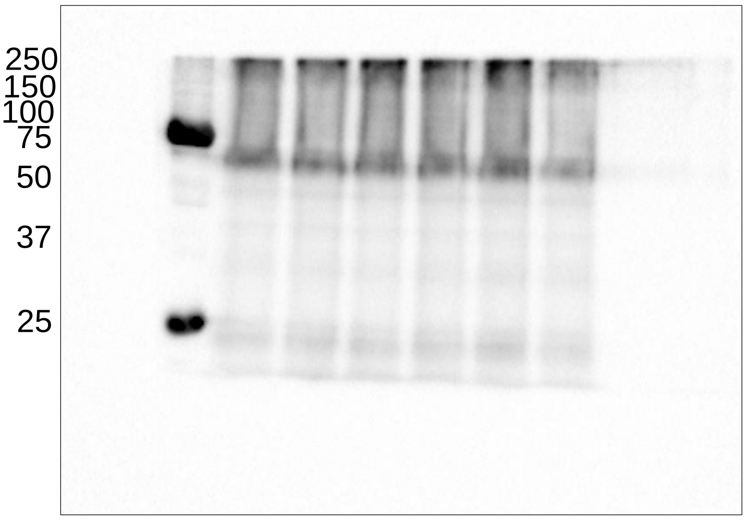

Exposure time 1.8 sec

WT WT WT KO KO KO

The original gels/blots images

Figure 3B for EAAC1 (EAAT3) 280, 57 and 48 kDa)

Full membrane

250
150
100
75
50
37

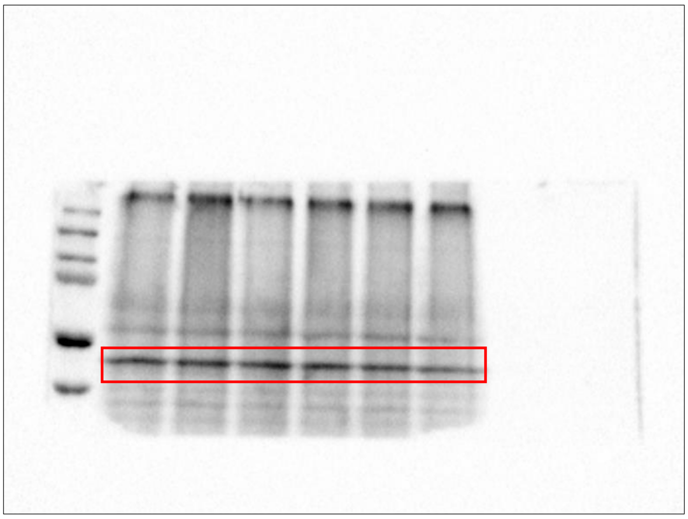

WT WT WT KO KO KO

Exposure time 10 sec

250
150
100
75
50
37

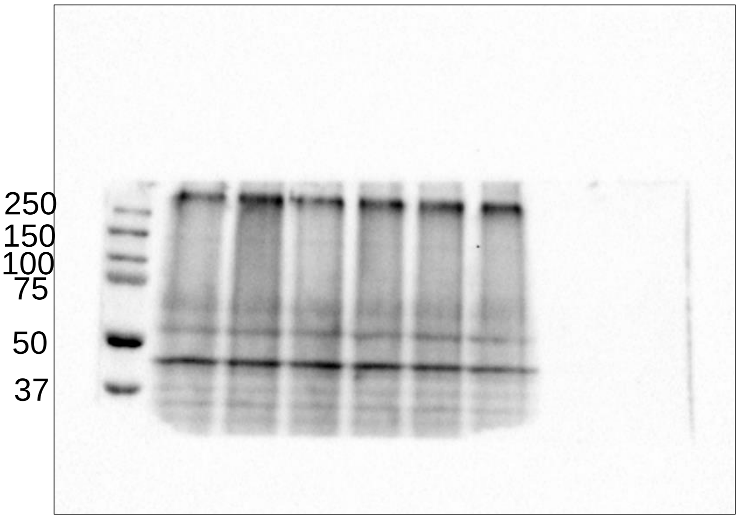

WT WT WT KO KO KO

Exposure time 15 sec

250
150
100
75
50
37

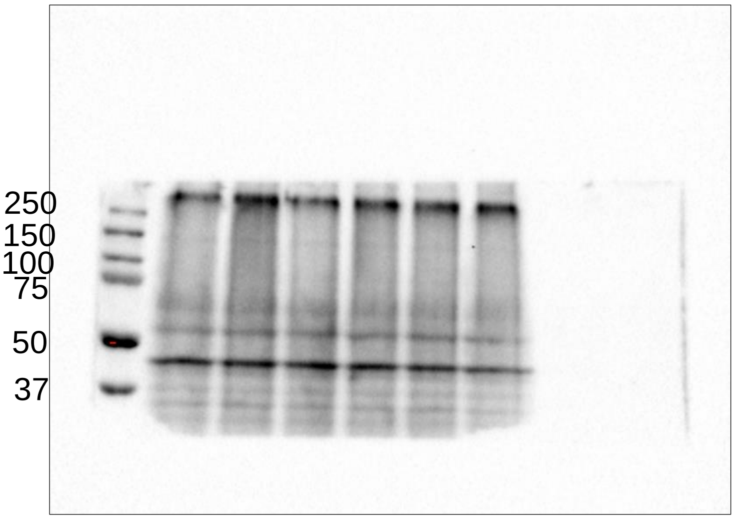

WT WT WT KO KO KO

Exposure time 20 sec

The original gels/blots images

Figure 3B for β-actin (42 kDa)

Full membrane

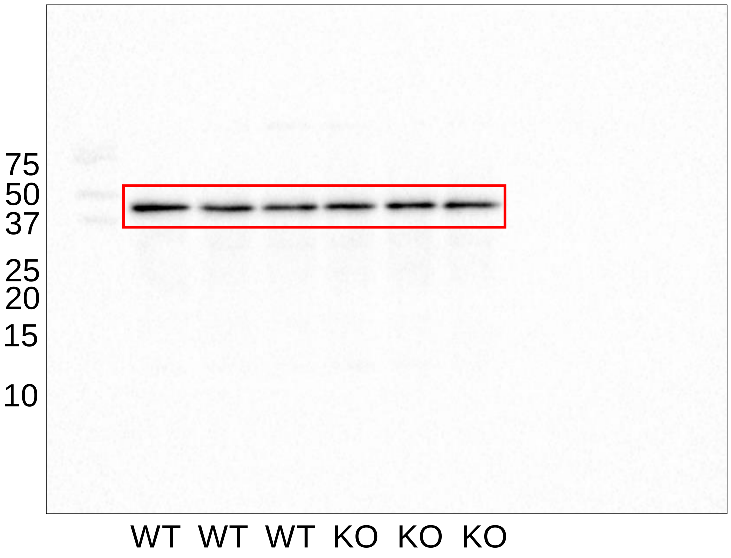

Exposure time 2 sec

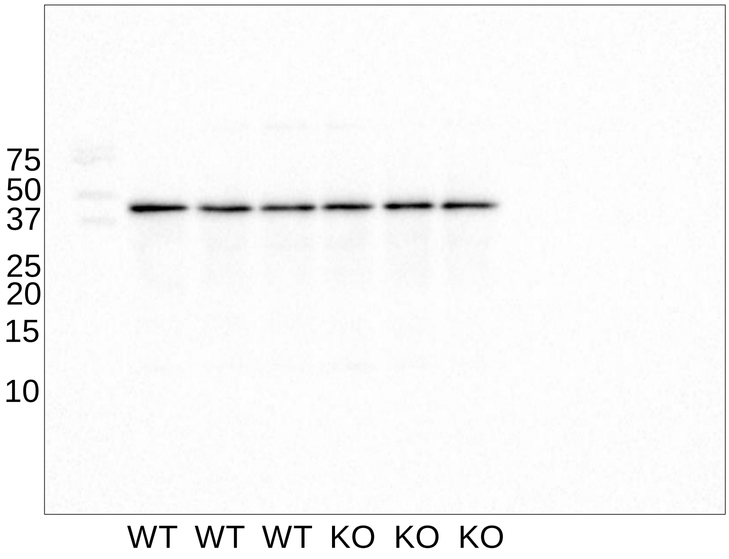

Exposure time 3 sec

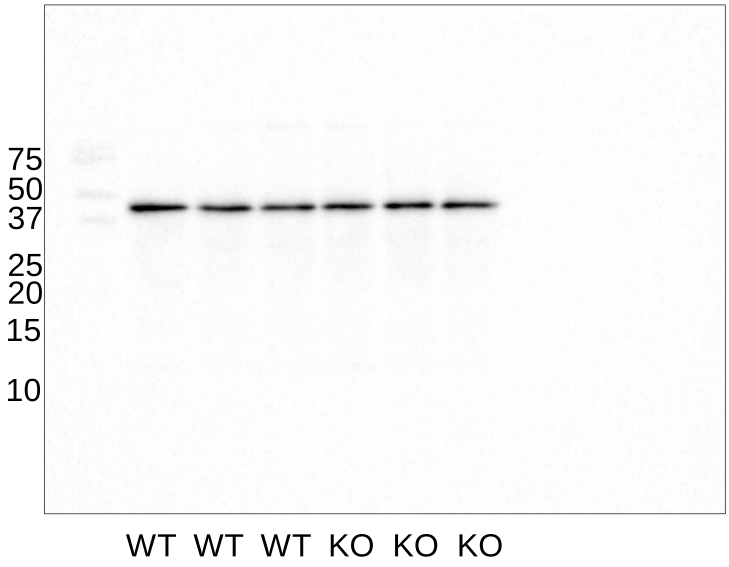

Exposure time 4 sec

The original gels/blots images

Figure 4E for Phospho-ERK1/2 (42 kDa)

Full membrane

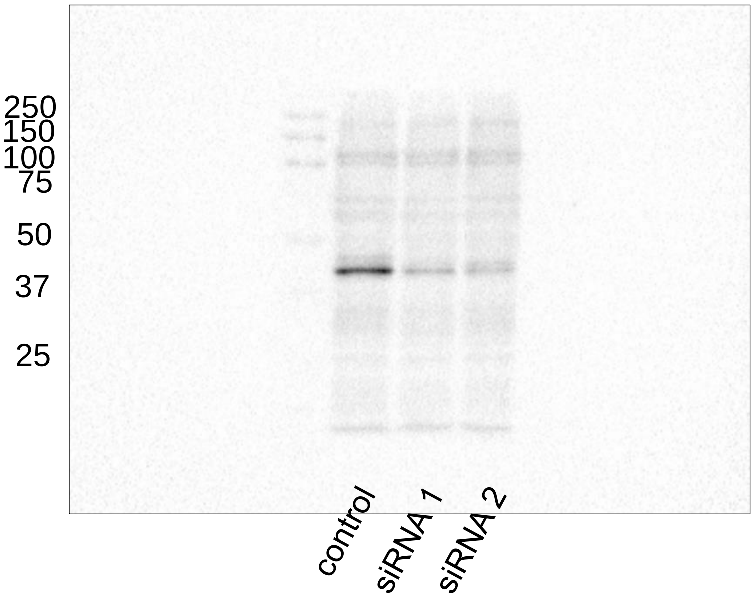

Exposure time 5 sec

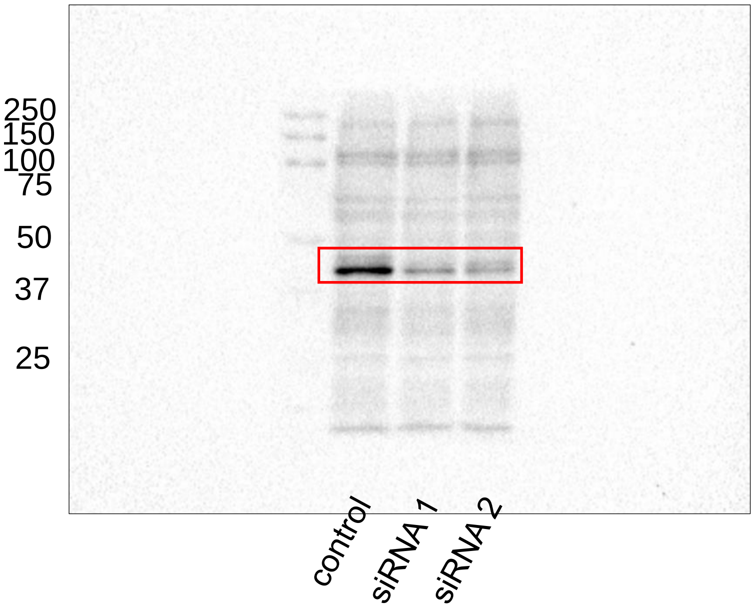

Exposure time 9 sec

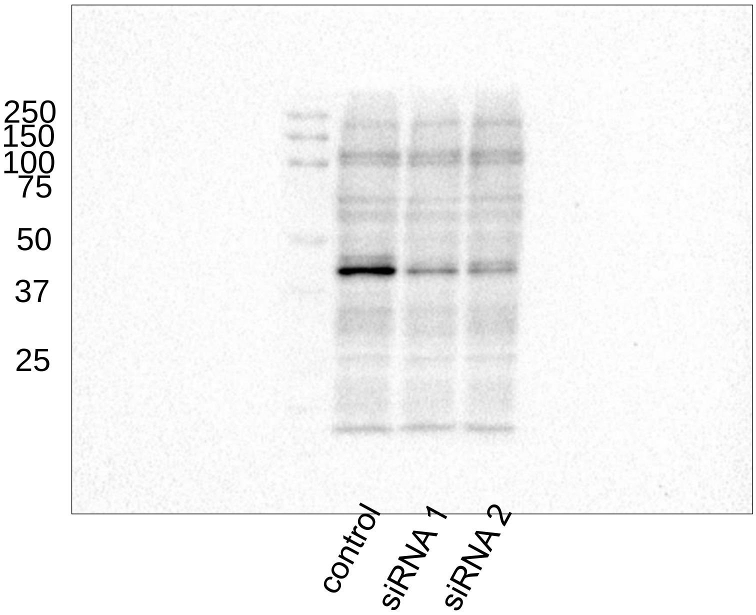

Exposure time 13 sec

The original gels/blots images

Figure 4E for ERK1/2 (44 and 42 kDa)

Full membrane

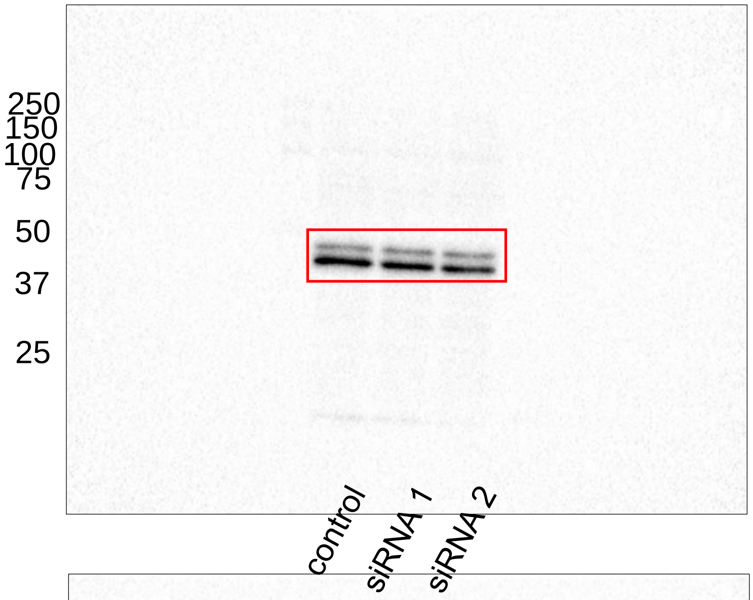

Exposure time 2 sec

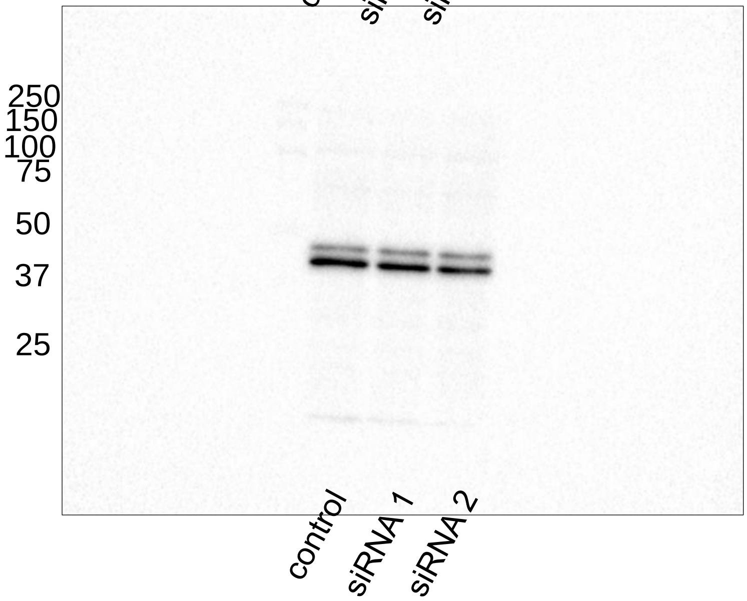

Exposure time 5 sec

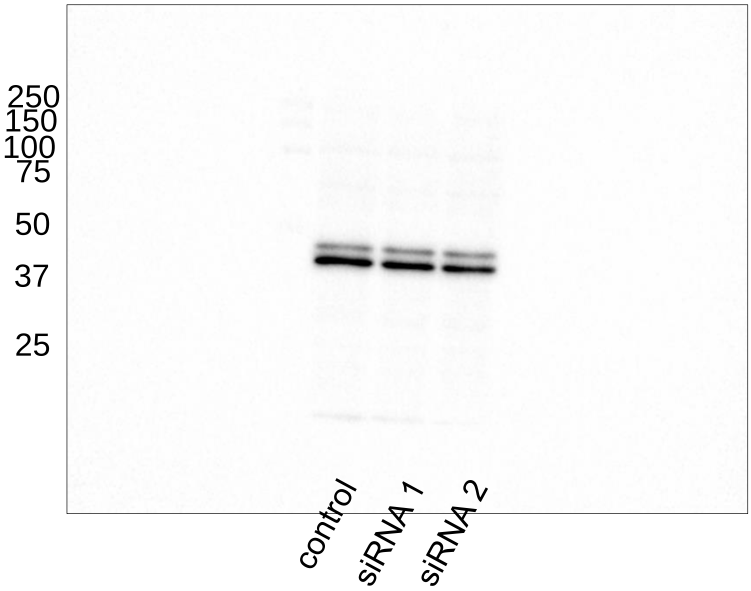

Exposure time 5 sec

The original gels/blots images

Figure 4E for β-actin (42 kDa)

Full membrane

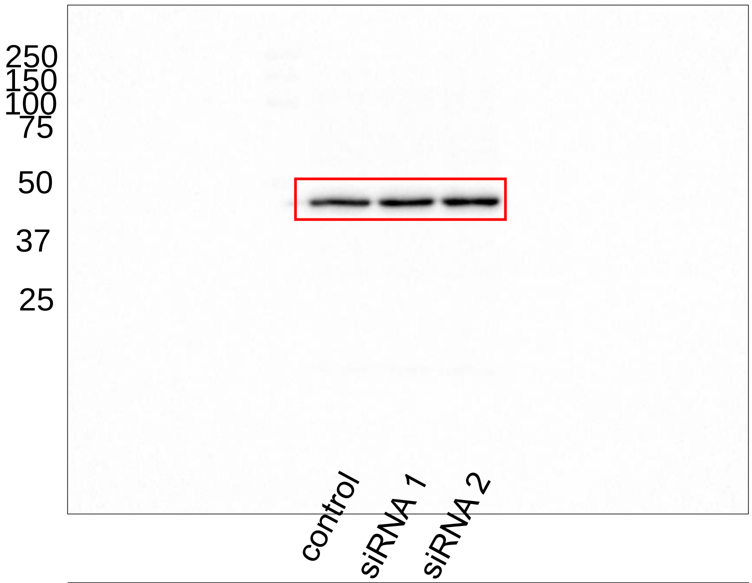

Exposure time 2 sec

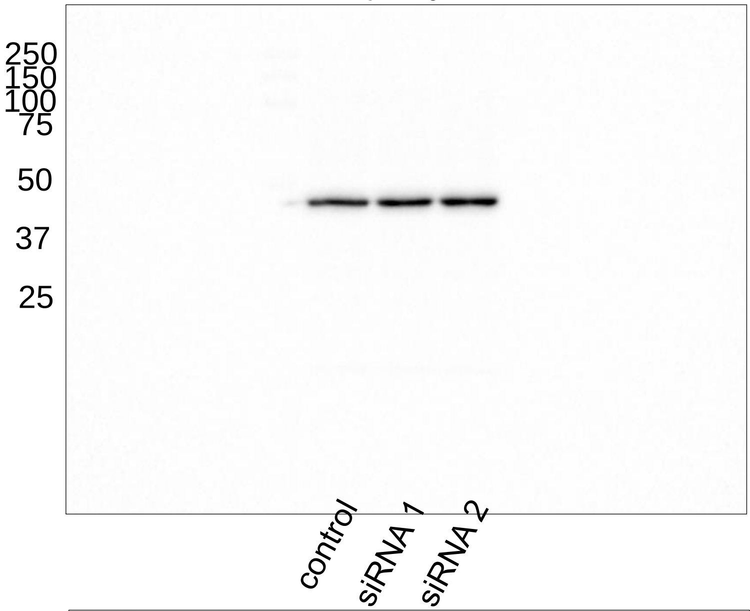

Exposure time 3 sec

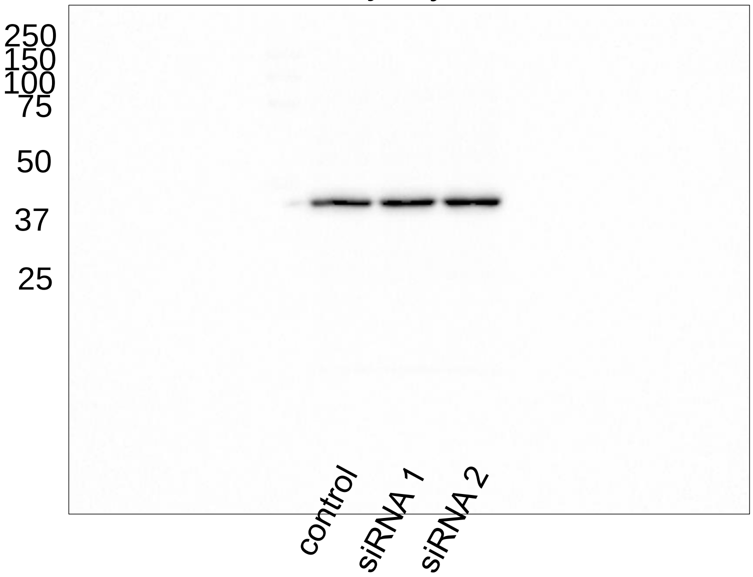

Exposure time 4 sec

The original gels/blots images

Supplemental Figure 2D for Ndufs4 (18 kDa)

Full membrane

WT KO

75
50
37
25
20
15
10

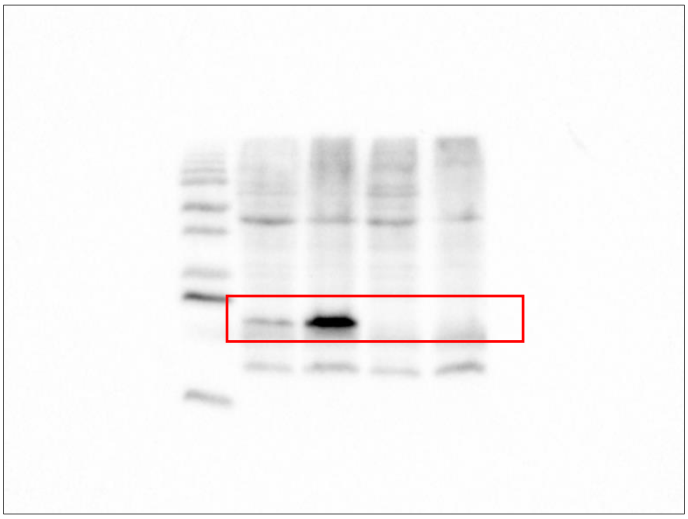

Exposure time 30 sec

T M T M

WT KO

75
50
37
25
20
15
10

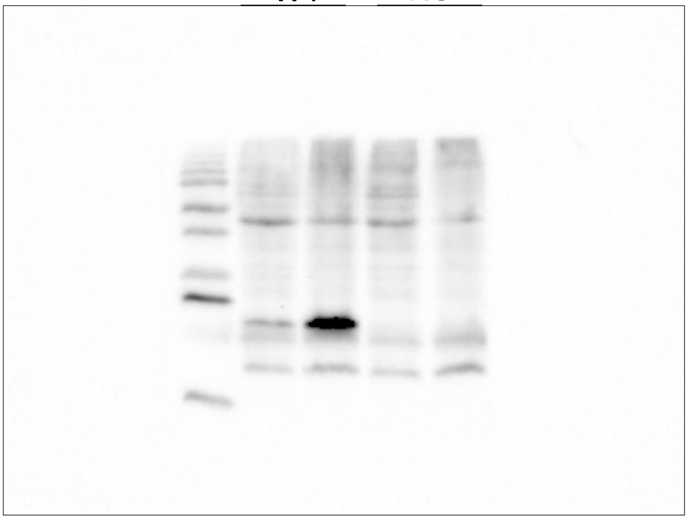

Exposure time 60 sec

T M T M

WT KO

75
50
37
25
20
15
10

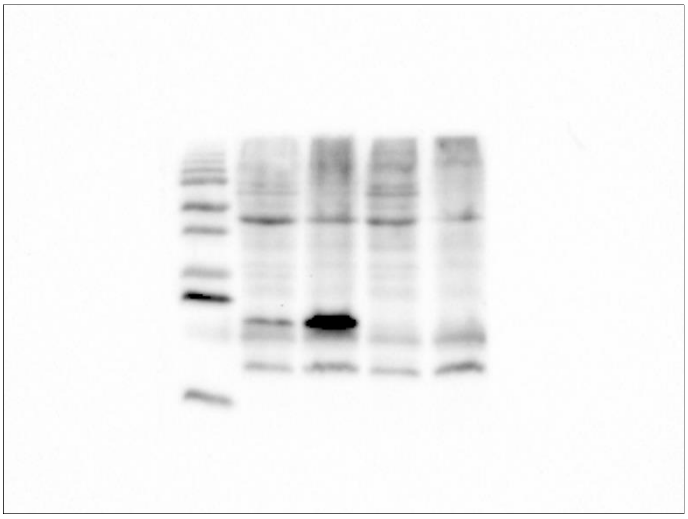

Exposure time 90 sec

T M T M

The original gels/blots images

Supplemental Figure 2D for VDAC1 (31 kDa)

Full membrane

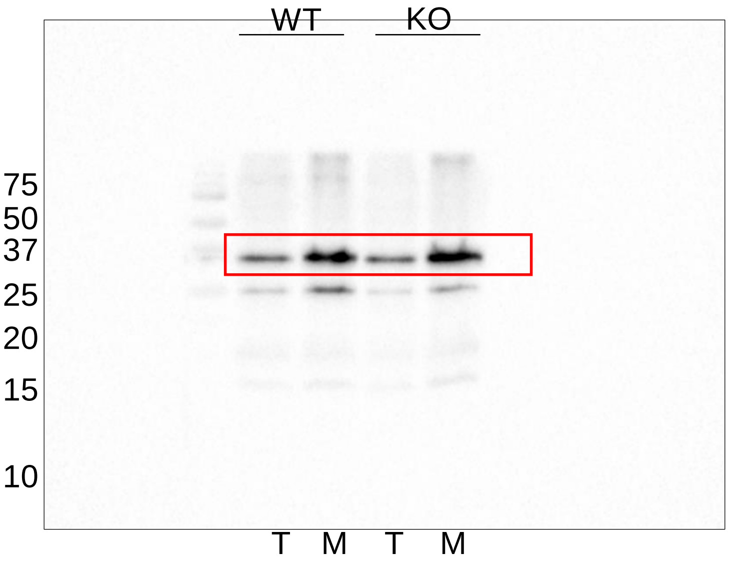

Exposure time 2 sec

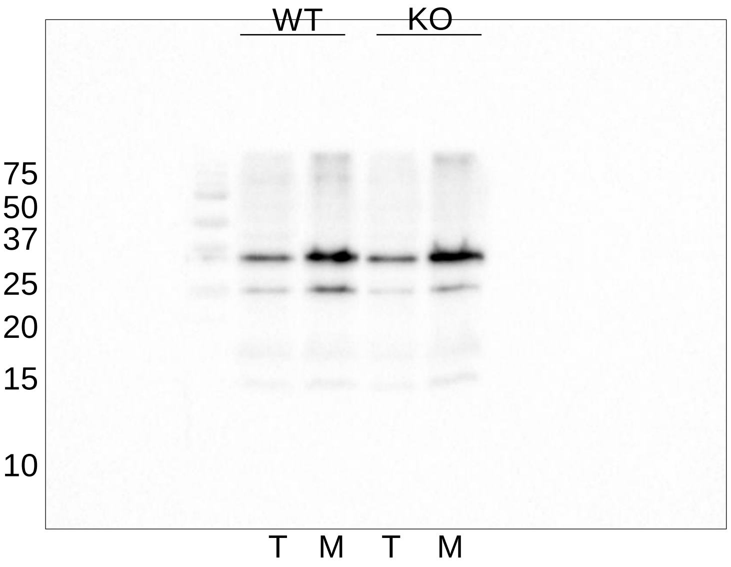

Exposure time 2.5 sec

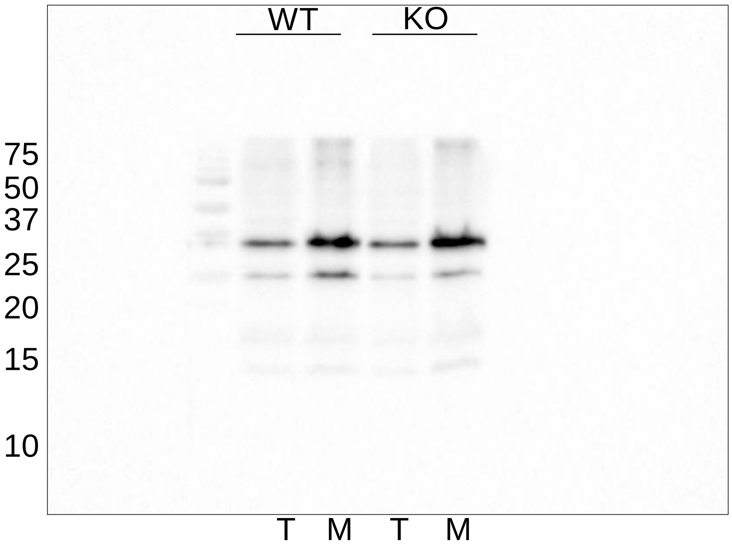

Exposure time 5 sec

The original gels/blots images

Supplemental Figure 4B for Ndufs4 (18 kDa)

Full membrane

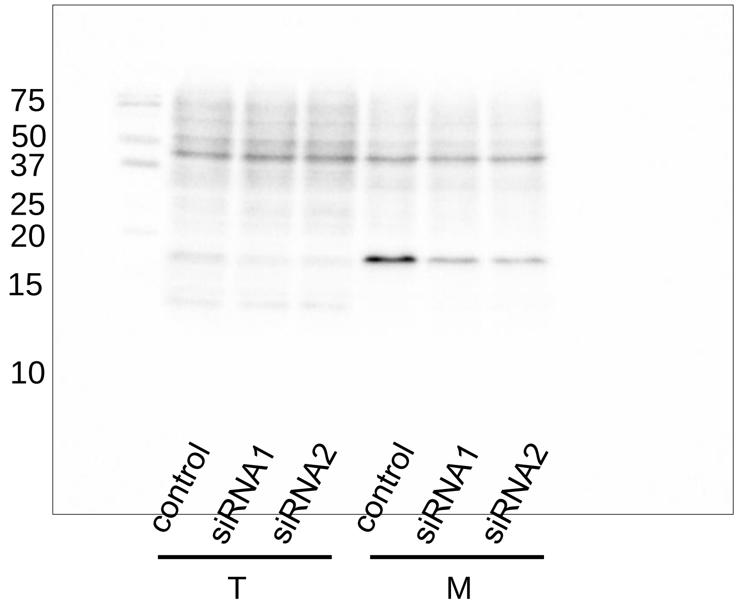

Exposure time 5 sec

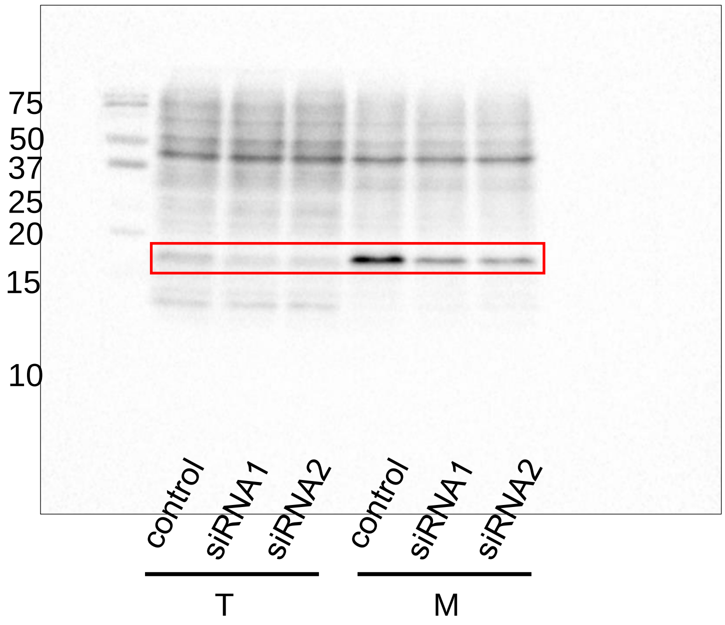

Exposure time 10 sec

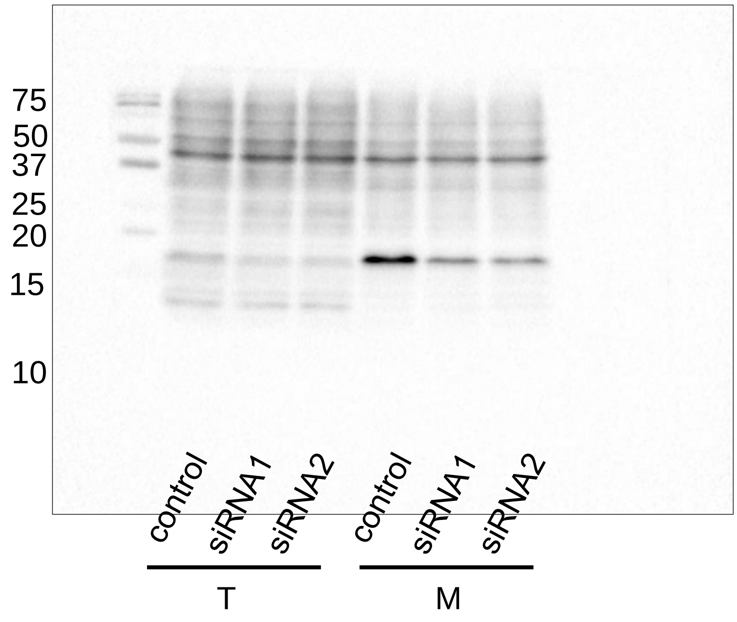

Exposure time 15 sec

The original gels/blots images

Supplemental Figure 4B for VDAC1 (31 kDa)

Full membrane

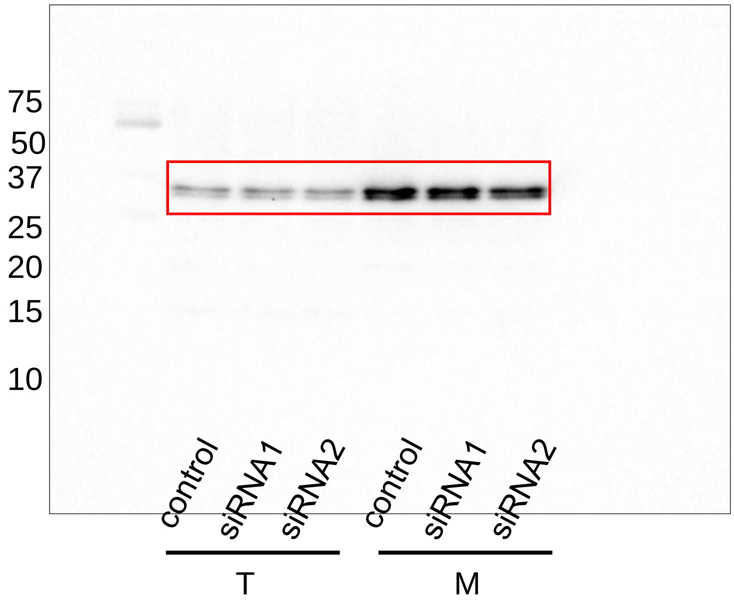

Exposure time 2 sec

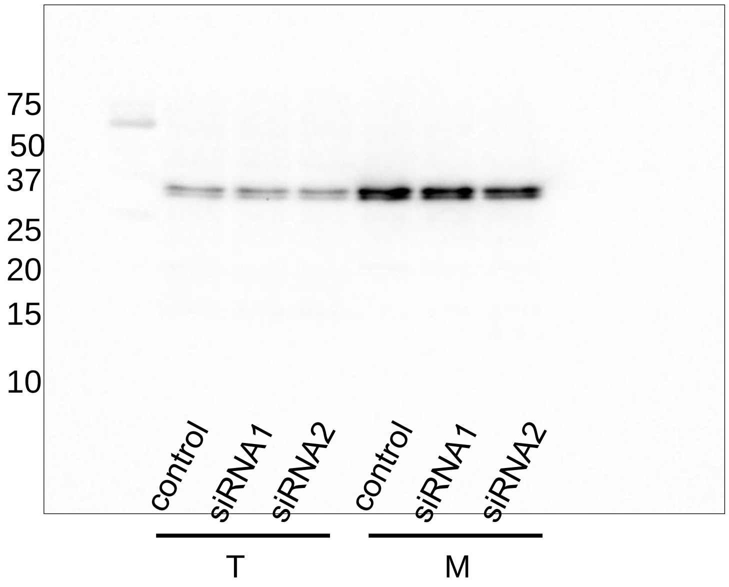

Exposure time 3 sec

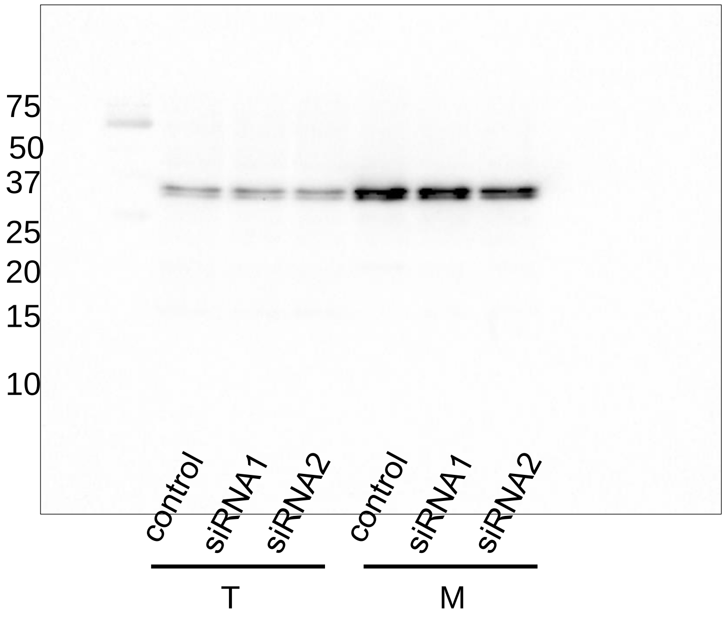

Exposure time 4 sec

The original gels/blots images

Supplemental Figure 4F for Phospho-ERK1/2 (42 kDa)

Full membrane

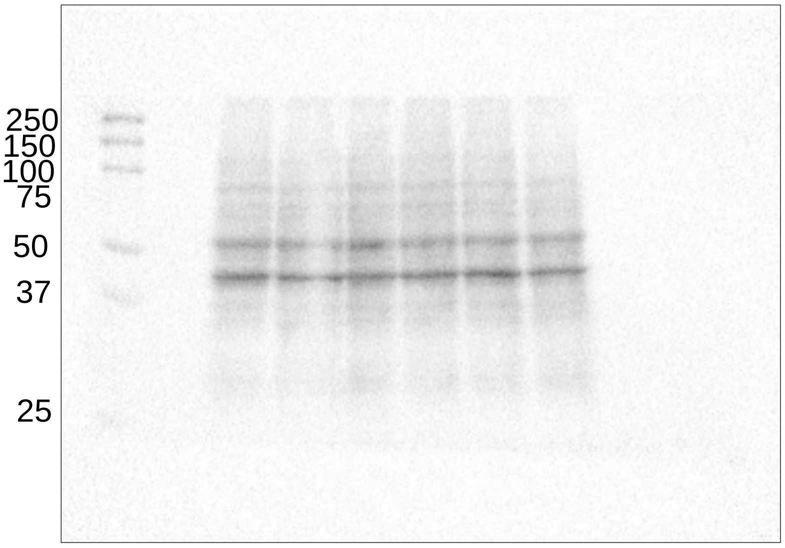

Exposure time 1 sec

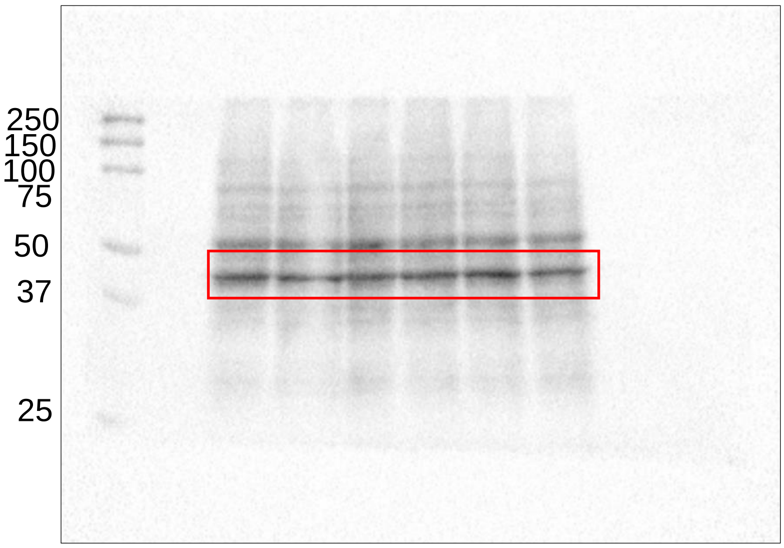

Exposure time 2 sec

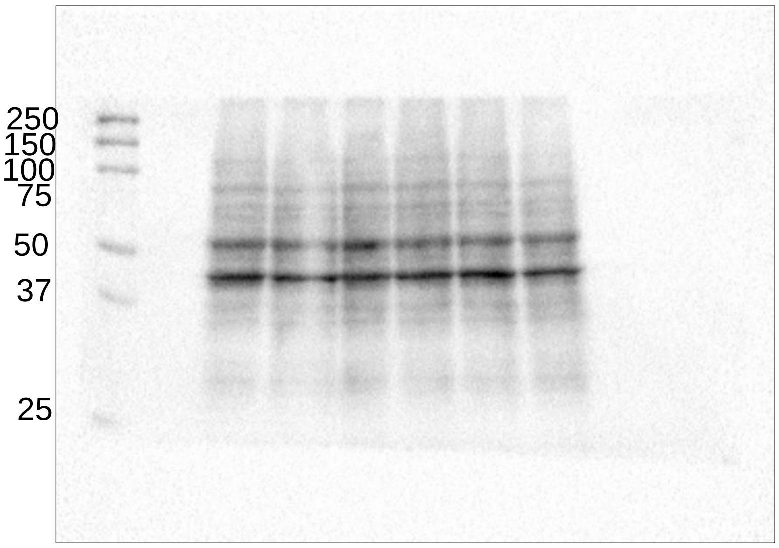

Exposure time 3 sec

The original gels/blots images

Supplemental Figure 4F for ERK1/2 (44 and 42 kDa)

Full membrane

250
150
100
75
50
37
25

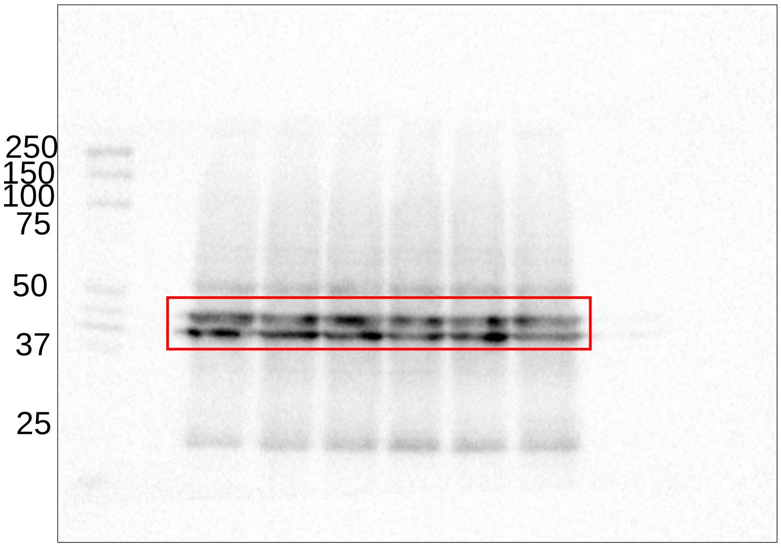

Exposure time 1 sec

WT WT WT KO KO KO

250
150
100
75
50
37
25

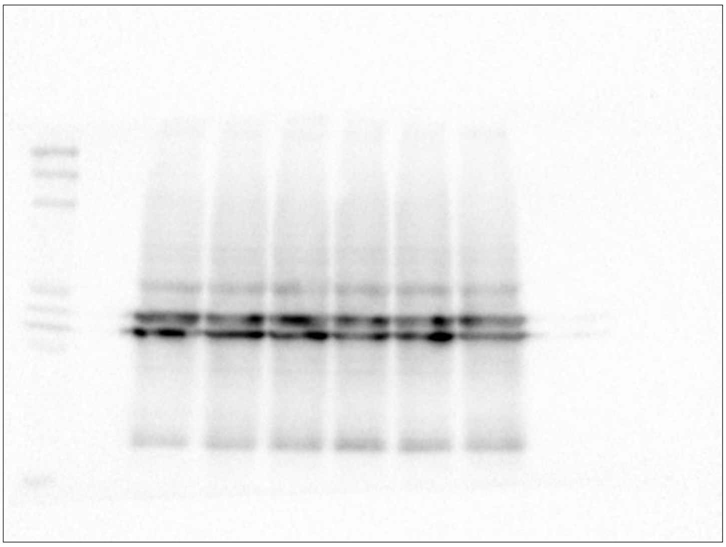

Exposure time 3 sec

WT WT WT KO KO KO

250
150
100
75
50
37
25

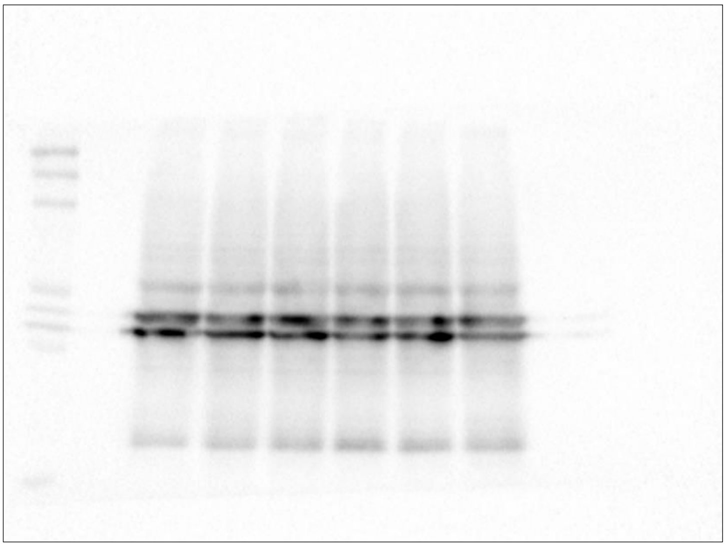

Exposure time 4 sec

WT WT WT KO KO KO

The original gels/blots images

Supplemental Figure 4F for β-actin (42 kDa)

Full membrane

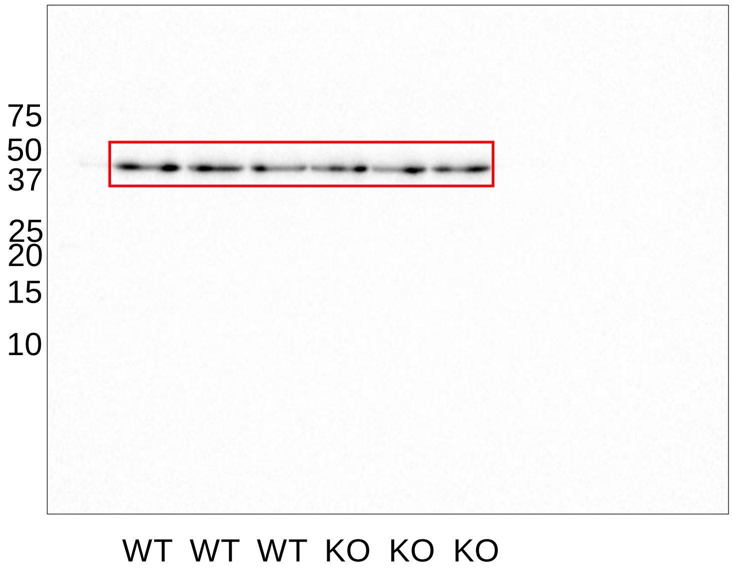

Exposure time 1 sec

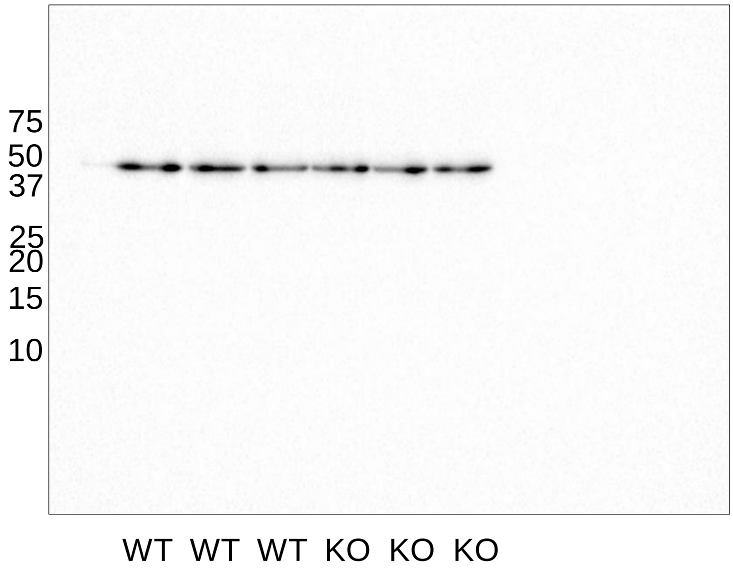

Exposure time 1.4 sec

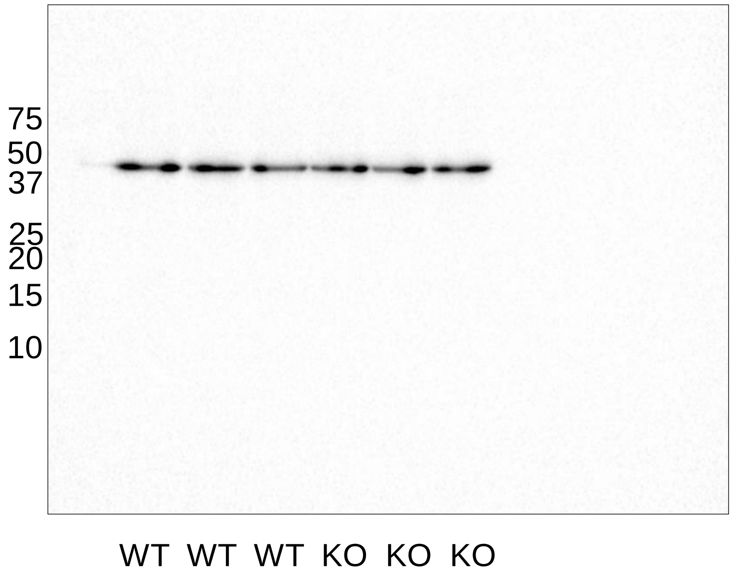

Exposure time 1.9 sec
